# Improving patients’ experiences of diagnosis and treatment of vertebral fracture: co-production of knowledge sharing resources

**DOI:** 10.1186/s12891-024-07281-9

**Published:** 2024-02-21

**Authors:** Sarah E. Bennett, Rachael Gooberman-Hill, Emma M. Clark, Zoe Paskins, Nicola Walsh, Sarah Drew

**Affiliations:** 1 Bristol Medical School, University of Bristol, Bristol, United Kingdom; 2 NIHR Bristol Biomedical Research Centre, Bristol, United Kingdom; 3 School of Medicine, Keele University, Keele, United Kingdom; 4 Haywood Academic Rheumatology Centre, Midlands Partnership University NHS Foundation Trust, Stoke-On-Trent, United Kingdom; 5 Centre for Health and Clinical Research, University of the West of England, Bristol, United Kingdom

**Keywords:** Qualitative, Osteoporosis, Vertebral fractures, Co-production

## Abstract

**Background:**

Osteoporosis involves changes to bones that makes them prone to fracture. The most common osteoporotic fracture is vertebral, in which one or more spinal vertebrae collapse. People with vertebral fracture are at high risk of further fractures, however around two-thirds remain undiagnosed. The National Institute for Health and Care Excellence (NICE) recommends bone protection therapies to reduce this risk. This study aimed to co-produce a range of knowledge sharing resources, for healthcare professionals in primary care and patients, to improve access to timely diagnosis and treatment.

**Methods:**

This study comprised three stages: 1. In-depth interviews with primary care healthcare professionals (*n* = 21) and patients with vertebral fractures (*n* = 24) to identify barriers and facilitators to diagnosis and treatment. 2. A taxonomy of barriers and facilitators to diagnosis were presented to three stakeholder groups (*n* = 18), who suggested ways of identifying, diagnosing and treating vertebral fractures. Fourteen recommendations were identified using the nominal group technique. 3. Two workshops were held with stakeholders to co-produce and refine the prototype knowledge sharing resources (*n* = 12).

**Results:**

Stage 1: Factors included lack of patient information about symptoms and risk factors, prioritisation of other conditions and use of self-management. Healthcare professionals felt vertebral fractures were harder to identify in lower risk groups and mistook them for other conditions. Difficulties in communication between primary and secondary care meant that patients were not always informed of their diagnosis, or did not start treatment promptly. Stage 2: 14 recommendations to improve management of vertebral fractures were identified, including for primary care healthcare professionals (*n* = 9) and patients (*n* = 5). Stage 3: The need for allied health professionals in primary care to be informed about vertebral fractures was highlighted, along with ensuring that resources appealed to under-represented groups. Prototype resources were developed. Changes included help-seeking guidance and clear explanations of medical language.

**Conclusions:**

The study used robust qualitative methods to co-produce knowledge sharing resources to improve diagnosis. A co-production approach enabled a focus on areas stakeholders thought to be beneficial to timely and accurate diagnosis and treatment. Dissemination of these resources to a range of stakeholders provides potential for substantial reach and spread.

**Supplementary Information:**

The online version contains supplementary material available at 10.1186/s12891-024-07281-9.

## Background

Osteoporotic bones are more prone to fracture. The most common osteoporotic fracture is a vertebral fracture which involves the breaking of a vertebrae in the spine, affecting 12% of older adults in Europe [1]. People with vertebral fractures are at high risk of further fractures, such as hip fracture, which have high rates of mortality [2]. The National Institute for Health and Care Excellence (NICE) recommends prescribing bone protection therapies to people who have experienced a fracture, to reduce the risk of further fractures by 30–50% [3]. However, it has been estimated that over two-thirds of people with vertebral fracture remain undiagnosed [1].

Vertebral fractures have several potential pathways to diagnosis. People may seek healthcare when they have symptoms that suggest they have experienced a vertebral fracture, such as height loss and back pain. Often, although not exclusively, people with symptoms present to primary care, and may be referred for clinically appropriate imaging such as spinal radiographs [4]. Vertebral fractures may also be identified opportunistically on images performed for other clinical indications. The Royal Osteoporosis Society has suggested that all images of the thoracic and/ or lumbar spine should be routinely evaluated for the presence of previously undiagnosed vertebral fractures [5].

Factors that act as barriers and facilitators to diagnosis are likely to be multi-faceted, from patient-related factors, such as the interpretation of symptoms and treatment-seeking behaviour to system-level factors, including the ability of clinicians to recognise the clinical presentation. Previous work has focused on clinicians’ experiences of diagnosis based on the understanding that they may benefit from tools to help them to identify clinical triggers that indicate whether a patient should be referred for spinal radiographs [5–7]. However, there are likely to be other barriers and facilitators that are as yet not known.

Improving ways to identify vertebral fractures and promote timely diagnosis and treatment requires support for healthcare professionals and patients. Support may include provision of information about how such fractures might present, how a diagnosis is made, and how symptoms should be managed. The process of co-creation enables knowledge sharing that can lead to appropriate resources [8, 9].

This study’s aim was to develop support for patients and professionals to enhance timely and accurate diagnosis and treatment. To achieve this, we: (1) identified barriers and facilitators to diagnosis and treatment; (2) developed a series of recommendations to identify osteoporotic vertebral fractures more effectively in the future; and (3) co-produced knowledge sharing resources for patients and healthcare professionals to help identify osteoporotic vertebral fractures. Improving identification of vertebral fractures is likely to be an important step in promoting access to treatment and subsequent fracture prevention.

## Methods

This development process used a three-phase qualitative process. These methods build on those that have been used to design information resources and interventions for healthcare professionals and patients for other musculoskeletal conditions. This includes work to develop a group-based intervention to support self-management of osteoporosis and low back pain (SOLAS) [10] and an intervention to support the self-management of fatigue for rheumatoid arthritis [11].

## Phase 1: In-depth interviews

To understand healthcare professionals’ and patients’ experiences and views of diagnosis, interviews with patients and healthcare professionals were conducted in parallel.

### Patients

Men and women ≥ 50 years old with a diagnosis of osteoporotic vertebral fracture were identified through two NHS secondary care sites from the south-west and west-midlands of England. These regions were chosen in order for patients from a range of backgrounds to be chosen, and with differing experiences and views. Levels of regional deprivation have been shown to influence help-seeking behaviours, with those from more deprived areas more likely to encounter barriers to help-seeking [12, 13]. Patients were identified by healthcare professionals working in relevant services and by reviewing clinical records. Patients were given an information pack with reply slip and invited to contact the study team, returning the reply slip in a stamped addressed envelope, or via email, if they were interested in taking part. Potential participants provided their contact information in the included reply slip, including their daytime and evening telephone numbers, email address, preferred time to be contacted, and their home address. Between two and three attempts were made to contact potential participants, usually by telephone but also by email if preferred. A question in the reply slip asked whether the prospective participant was aware that they have an osteoporotic vertebral fracture. Only those who were aware of their fracture were eligible to participate in the study. Sometimes, patients with osteoporotic vertebral fractures can be unaware of their diagnosis. For example, in a review of 459 chest x-rays, 40% of patients with an osteoporotic vertebral fracture did not receive a correct diagnosis on the radiography report [14]. Similarly, in a survey of women in a national claims database, over half of those surveyed (54%) had not been told of their osteoporosis diagnosis [15]. In order to avoid contacting patients who were unaware of their diagnosis, the first question in the reply slip asked recipients “Have you ever been told by a healthcare professional that you have broken a bone in your spine (vertebral fracture)?” with the option to tick “yes” or “no”. By employing this method, rather than calling or contacting participants directly, this sought to mitigate any potential surprise or distress, and avoid participants finding out details about their health of which they were previously unaware [16]. Only those who were aware of their fracture were recruited into the study, in order to avoid any potential distress.

Participants were all ≥ 50 years old to maximise inclusion of participants with vertebral fractures relating to osteoporosis rather than other pathology (e.g. traumatic) [17]. A total of 100 participant information packs were distributed and 33 patients expressed an interest in participating. Of these, 24 took part. The remainder either could not be reached or were unaware that they had a vertebral fracture. Final sample size was determined during analysis, where we found that the specificity of the sample and the study aims, as well as the depth of the information in relation to the aims meant that the data collected contained sufficient information [18].

Telephone interviews lasting 17 to 90 min (mean = 39 min) were conducted using a topic guide [19]. Interviews explored patients’ diagnostic journeys, including experiences of fracture, treatment-seeking behaviour, experiences accessing services and barriers or facilitators to diagnosis. The topic guide was devised in collaboration with a patient representative living with vertebral fractures. Interviews were carried out by the first author (SEB), a female postdoctoral qualitative researcher who has several years of experience in conduct of qualitative research interviews with under-researched and vulnerable populations.

### Healthcare professionals

Healthcare professionals in primary care with experience diagnosing osteoporotic vertebral fractures were recruited from across England. Adverts were disseminated on social media by two Local Clinical Research Networks (LCRNs), clinical and academic science networks. A total of 34 healthcare professionals contacted the research team and of these, 21 participated. Final sample size was determined when information power was achieved [18].

Telephone interviews lasting 17 to 39 min (mean = 30 min) were carried out using a topic guide [19]. Interviews explored their understanding of vertebral fractures, experiences of diagnosis, perceived barriers and facilitators to identification, and referral for imaging to confirm diagnosis.

### Analysis

Interviews were audio-recorded, transcribed, anonymised, and imported into NVivo12 qualitative data management software. An inductive thematic approach was used to identify barriers and facilitators to diagnosis and treatment [20]. Analysis developed a taxonomy of barriers and facilitators that were grouped into four stages of the Model of Pathways to Treatment care pathway [21]. Two transcripts from interviews with healthcare professionals and two from patients were independently coded by two researchers, reviewed and refined to reach an agreed code list that was applied and refined further with agreement from both researchers as analysis progressed [20]. Based on analysis, we added an additional stage to the Model of Pathways to Treatment, ‘communication of diagnosis’, since this was identified as a key element of the process that patients encountered, and that impacted on time to treatment. More detailed findings relating to barriers and facilitators to diagnosis and treatment will be presented in another article.

## Phase 2: developing recommendations

Phase 2 comprised stakeholder group meetings to develop recommendations to improve identification of people with osteoporotic vertebral fractures, based on barriers and facilitators identified in Phase 1.

Stakeholder groups included patients and healthcare professionals involved in prevention and diagnosis of fragility fractures in primary and secondary care. Healthcare professionals in primary care were recruited as above. Healthcare professionals involved in the prevention of fragility fractures in secondary care were identified through study sites identified in Phase 1. Patients were identified through patient advocacy groups and participants in previous research who had consented to be contacted for future research. In total 191 participant information packs were either posted or emailed to patients and of these, 14 patients and carers expressed an interest in participating, of which 12 took part. A total of 7 healthcare professionals contacted the research team to express an interest in taking part and of these 6 participated.

### Nominal group technique

**Fig. 1:**
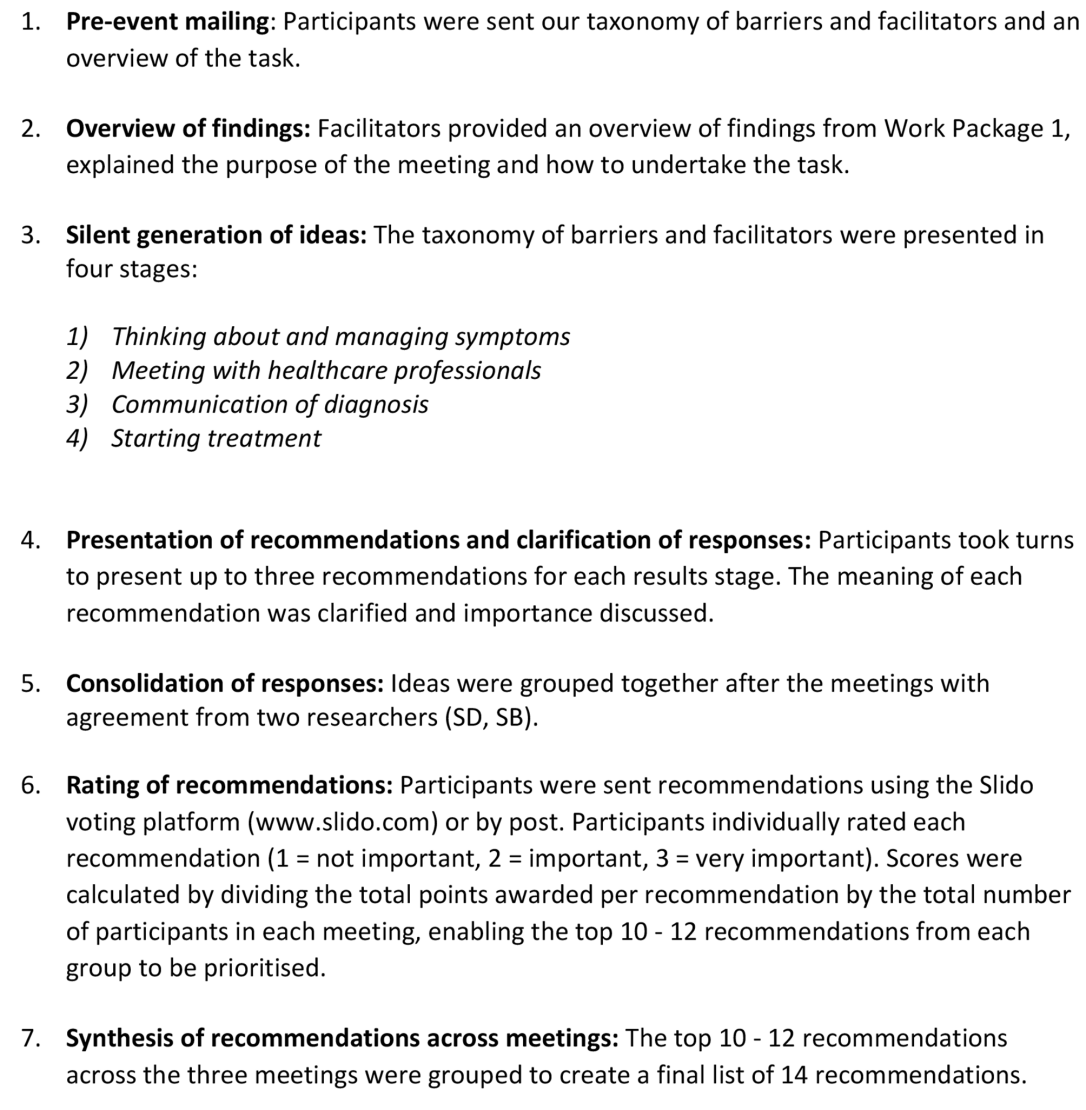
Stages of the nominal group technique

Recommendations were developed using the Nominal Group Technique [22] where consolidation of responses and rating of recommendations took place after the meetings enabling remote participation. Recommendations were also synthesised across three groups which allowed us to increase our sample size and include a wider range of participants. The NGT method has been used to facilitate the development and implementation of new resources. Examples include elicitation of healthcare professionals’ input into an osteoporosis quality improvement intervention [23], interventions for hip or knee osteoarthritis [24] and development of prototype exercise machines [25]. NGT has also been used to support identification of research priorities, for instance relating to the use of bisphosphonates in the UK [26]. Two meetings were held online using Zoom (Groups 1 and 2) and teleconference (Group 3). The meetings comprised 7 stages (see Fig. 1 below).SB and SD performed the analysis, and the early interpretations were explored and agreed with another member of the research team. The 14 top ranked recommendations were used to inform the resources generated in Phase 3.

## Phase 3: co-production of resources

We used a Communities of Practice (CoP) approach: a collection of people who share a common interest who interact to problem-solve and share knowledge [27]. The CoP was chosen as this method involves bringing a range of stakeholders together in partnership [28, 29], to design services [30], interventions [31–33] or health literature [34, 35]. Experience-based co-design, by means of engaging healthcare professionals and patients in the improvement of care pathways is key to improving both patient care, and healthcare professionals experiences of care [36, 37]. The benefits of the CoP method can be wide-ranging, enabling members to learn from mutual experiences, and to solve problems within healthcare [38].

The CoP included patients with osteoporotic vertebral fractures, their partners, healthcare professionals involved in the diagnosis of vertebral fractures in primary and secondary care and a representative from an osteoporosis patient support group. Participants were recruited using the strategies outlined in Phase 2.

Co-production was achieved in three stages:

### Stage 1: idea generation

A 1.5 h idea generation workshop was held using video teleconferencing. A researcher facilitated a guided discussion to explore which healthcare professionals and/or patients could benefit from a knowledge sharing resource, at what time-points in the care pathway, different types of resources, and targeted dissemination strategies. To design the structure, participants undertook a card-sorting task using Miro, an online co-creation tool (www.miro.com). To support interaction with Miro, rather than making the Miro platform available to all users individually, the co-convenor of the session (SB) shared their own interaction with Miro, taking and entering suggestions and direction from participants. Participants were presented with a list of topics on cards that formed the content of the resource, grouping and labelling them in a way that made sense to them [39]. The workshop was audio-recorded, transcribed and data analysed using a descriptive thematic approach to summarise themes and reflections on the task [40].

### Stage 2: development of prototypes

Based on workshop findings, prototype leaflets and posters were designed. Information was provided in a question-and-answer structure. Content was developed iteratively within the research team with input from an osteoporosis patient support group. An infographic was developed to allow messages to be conveyed graphically.

### Stage 3: Consultation

A second workshop refined the resources using video teleconferencing and lasted 1.5 h. Prototype resources were evaluated in relation to their (i) acceptability: the extent to which participants judge the prototype resources as appropriate for their intended purpose; (ii) adequacy: the sufficiency of the prototype information resources; (iii) tone: the attitude towards the reader, and; (iv) readability: how easy the text is to read and understand. Prototype resources were provided in advance of the meeting, and were explored in-depth during the meeting. We allowed time for participants to view them before and during the meetings including through the facilitators reading out the text that was included in resources. The workshop was audio-recorded and transcribed, the data reviewed line-by-line to identify potential changes.

## Results

### Phase 1: In-depth interviews

#### Participant characteristics

A total of 43 participants took part in interviews. Participant characteristics are shown in Tables 1 and 2. All names are pseudonyms. First Contact Physiotherapists (FCPs) are advanced practitioners who have extensive experience in the assessment, diagnosis and management of musculoskeletal (MSK) conditions [41]. Rather than visiting a GP, patients with musculoskeletal pain typically book to see a FCP via GP receptionists [42], acting as a first point of contact for patients with MSK pain. The role was developed to ensure that patients in the UK have faster access to the most effective care, without needing to visit hospital-based physiotherapy services [41].

**Table 1:** Participants in Phase 1: In-depth interviews with patients

Pseudonym	Age range	Sex (M/F)	Ethnicity	Number of vertebral fractures	Bone health (osteoporosis or any other fractures)	Other conditions	Hospital Site pseudonym
Alice	76–80	F	White	4 (but unsure)	Osteoporosis	Rheumatoid arthritis Lung disease (unspecified)	Merryfield
Grace	70–74	F	White	1	Osteoporosis Wrist fracture	Epilepsy	Merryfield
Harriet	75–79	F	White	2	Osteoporosis Femur fracture	Rheumatoid arthritis Lung disease (unspecified)	Merryfield
Alexandra	70–74	F	White	1	Osteopenia	Diabetes (Type 1)	Merryfield
Mary	75–79	F	White	3	Osteoporosis Humeral fracture	Hiatus hernia	Merryfield
Kirsten	65–69	F	White	2	Osteoporosis	Hypothyroidism	Merryfield
Jane	65–69	F	White	1 (but unsure)	Osteoporosis	Rheumatoid arthritis Asthma	Merryfield
Iris	75–79	F	White	3	Osteoporosis	None	Merryfield
Georgia	70–74	F	White	1	Osteoporosis Wrist fracture	None	Merryfield
Ruth	75–79	F	White	1	Osteoporosis Wrist fracture Shoulder fracture	Heart problems (unspecified)	Merryfield
Beth	65–69	F	White	1	Osteoporosis Metatarsal fractures	Asthma History of cancer (kidney)	Merryfield
Susan	65–69	F	White	3 (but unsure)	Osteoporosis Ankle fracture Wrist fracture		Merryfield
Sam	80–84	M	White	3 or 4 (but unsure)	None	Hiatus hernia	Merryfield
Martin	55–59	M	White	1	None	None	Merryfield
Phillip	55–59	M	White	1	Osteoporosis Finger fracture Wrist fracture Rib fracture	Osteoarthritis Migraines	Easterhill
Anthony	75–79	M	White	(Unsure)	Arm fracture Collarbone fracture Nose fracture	Hiatus hernia	Easterhill
David	65–69	M	White	2 (but unsure)	None	Bowel cancer	Easterhill
Anne	70–74	F	White	2	Osteoporosis Ankle fracture Wrist fracture Foot fracture	Chronic Obstructive Pulmonary Disease (COPD) Heart failure	Easterhill
Heidi	80–84	F	White	1	Osteoporosis	Arthritis (unspecified)	Easterhill
Lucy	70–74	F	White	2 (but unsure)	Foot fracture	Diabetes (Type 2)	Easterhill
Claire	65–69	F	White	1	Osteoporosis Ankle fracture Toe fracture Finger fracture	Rheumatoid Arthritis Emphysema Knee replacement	Easterhill
Olivia	60–64	F	White	5 (but unsure)	Osteoporosis Elbow fracture Foot fracture	Emphysema Fibromyalgia Post-Traumatic Stress Disorder (PTSD)	Easterhill
Hannah	75–79	F	White	2	Osteoporosis Rib fracture	Hypertension	Easterhill
Noah	75–79	M	White	5	Osteoporosis	Bladder cancer Plantar fasciitis	Easterhill

Acronyms: F = female, M = male

**Table 2:** Participants recruited to Phase 1: In-depth interviews with primary care professionals

Pseudonym	Age range	Sex (M/F)	Role
Akal	45–49	M	General Practitioner
Emma	45–49	F	First Contact Physiotherapist
Charlotte	55–59	F	Advanced Physiotherapy Practitioner
Amelia	55–59	F	First Contact Physiotherapist
Dylan	35–39	M	First Contact Physiotherapist
Rory	40–44	M	First Contact Physiotherapist
Ava	50–54	F	First Contact Physiotherapist
Sophie	40–44	F	General Practitioner
Isabelle	45–49	F	General Practitioner
May	30–34	F	General Practitioner
Evelyn	45–49	F	General Practitioner
Abigail	35–39	F	Physiotherapist
Chloe	40–44	F	First Contact Physiotherapist
Daniel	45–49	M	First Contact Physiotherapist
Hazel	45–49	F	Musculoskeletal Physiotherapist
Adam	55–59	M	General Practitioner
Ethan	40–44	M	First Contact Physiotherapist
Grace	35–39	F	First Contact Physiotherapist
Leah	60–64	F	General Practitioner

Acronyms: F = female, M = male,

### Findings

Barriers and facilitators to diagnosis and treatment were grouped into four stages: (i) patient appraisal, self-management and decision to consult healthcare professional; (ii) healthcare professional appraisal, investigations, referrals and appointments; (iii) communication of diagnosis; and (iv) planning and scheduling of treatment. Table 3 provides the taxonomy of barriers and facilitators with illustrative quotations.

**Table 3:** Taxonomy of barriers and facilitators to diagnosis and treatment initiation

Patient appraisal, self-management and decision to consult healthcare professional		
Facilitators	Barriers	Illustrative quotations
Patients experience pain that is severe and different to any pain they have experienced before. Patients’ back pain doesn’t get better with time. Other people noticed the symptoms and encouraged them to visit healthcare professionals. Patients talk to other people about the pain. Other people include friends or family who encourage them to visit a healthcare professional. Patients associate their pain with an injury such as a fall.	Patients do not know what a vertebral fracture is and what the symptoms are. Patients living with osteoporosis are not aware that they are at risk of vertebral fractures. Patients do not think the pain is serious. Patients mistake the pain for another issue such as a kidney infection, arthritis, broken rib or muscular pain. Patients have a vertebral fracture that doesn’t have any symptoms. Patients do not associate their pain with an injury such as a fall. Patients choose to self-manage symptoms. Patients don’t think getting help for their back pain is important and prioritises other conditions or feels that they are too busy. Patients feel that they do not want to ‘bother’ healthcare professionals or ‘make a fuss’. Patients feel that their healthcare professional does not take their pain seriously and do not want to re-visit them for help if their pain doesn’t improve.	“It did cross my mind that I was just being a big baby and the pain was not as bad as what I thought it was, if you know what I mean.” [Olivia, patient with vertebral fractures] “I wouldn’t have known what to recognise actually to be quite honest, no I wouldn’t. If I had something wrong with my back I’d just [think I] pulled something or done something. [Anthony, patient with vertebral fractures] “I was taking so many tablets I think I was overdosing. I was putting Deep Heat, Ibuprofen and heat patches on because as I say, I didn’t know what it was. And I was taking eight tramadol a day, four amitriptyline, eight paracetamol and I was just going round the bend I reckon.” [Beth, patient with vertebral fractures]] “I’ve had quite a lot of pain with prolapsed discs and all sorts of things but [the vertebral fracture] was different pain” [Anthony,, patient with vertebral fractures] “I have a friend…he said ‘You look like a hunchback!’ and I thought ‘Well, that’s a bit cruel!’” [Alice, patient with vertebral fractures] “They [healthcare professionals] didn’t seem to be particularly bothered [about my back pain] and I was made to feel like I was just being a nuisance basically.” [Claire]
**Healthcare professional appraisal, investigations, referrals and appointments**		
**Facilitators**	**Barriers**	**Illustrative quotations**
Healthcare professionals in general practice are aware of risk factors for vertebral fractures such as age, sex, and low BMI. Healthcare professionals in general practice have knowledge of the symptoms of vertebral fracture such as height loss and severe back pain. Knowledge of referral pathways to request imaging to confirm diagnosis and to specialists in hospital for assessment. Suspicion of vertebral fracture is clearly described on radiology request. Diagnosis of vertebral fracture clearly and unambiguously indicated on the imaging report. Healthcare professionals in general practice refer patients to A&E to speed up access to imaging.	Healthcare professionals in general practice and Accident and Emergency (A&E) mistake the symptoms of vertebral fractures for other conditions or causes such as a pulled muscle or broken rib. Healthcare professionals in general practice tell patients to ‘wait and see’ if their vertebral fracture symptoms get better on their own before initiating further investigations. Healthcare professionals find vertebral fractures harder to identify in men and young people because they are less likely to be at risk. Healthcare professionals find it more difficult to identify vertebral fractures in patients who do not present with severe symptoms. Healthcare professionals are discouraged from routinely imaging patients who present with low back pain due to NICE guidelines. Lack of incentive to identify osteoporosis in primary care through reimbursement schemes.	“I don’t think [diagnosing vertebral fractures is] as straightforward… males do get osteoporosis. So it’s always on your differential diagnosis… I’d say [diagnosis is] moderately difficult.” [Ava, FCP] “Sometimes it’s acute and clear cut that yes someone’s had a sort of sudden collapse, sometimes it’s a more of a gradual crumble I imagine.” [Sophie, GP] “What I tend to find…Is just that severity of pain and their inability to straighten up… They really struggle standing and straightening up” [Emma, FCP] “We’re encouraged not to image people’s backs so you know we never, hardly ever send people for [spine] x-rays … we’re told not to do that.” [Isabelle, GP]
**Communication of diagnosis**		
**Facilitators**	**Barriers**	**Illustrative quotations**
A diagnosis of vertebral fracture is clearly communicated to patients either verbally by their GP or specialist in hospital. Patients are given information about vertebral fractures and osteoporosis when they are diagnosed to help them understand what they are and how to manage them. Patients provided with clarity on how their vertebral fracture was diagnosed and how many they have sustained. Patients informed in writing are provided with a patient friendly letter. Healthcare professionals explain what a vertebral fracture is to help reduce the feelings of shock and surprise when they are diagnosed. Clarity on which healthcare professional should be informing patients about their vertebral fracture, preferably by referring clinician.	Patients find out about their diagnosis by being copied into medical letters and find some of the ‘big words’ confusing and difficult to understand as they are not explained. Healthcare professionals use confusing words to inform patients that they have had a vertebral fracture such as ‘compression fracture’ or ‘wedge deformity’. Patients are therefore unsure if they have had a vertebral fracture. Patients find the term “vertebral fractures” alarming as it makes it sound like they have had a catastrophic injury. Patients are not clearly told how many vertebral fractures they have had. Healthcare professionals are unclear if a patient has been told about their vertebral fracture as they are being managed by healthcare professionals in hospital and at their GP surgery. Some patients are therefore not informed.	“It’s just the terminology that’s maybe used in telling the patient what the, it can be very, very scary if a patient is told they’ve got a fracture in their spine. And it’s just sometimes the way it’s relayed. So that’s why I always try and follow up my own x-ray requests.” [Amelia, FCP] “[The Fracture Liaison Service reviewed images and] picked up some fractures and then we’re at the stage where we don’t know if they’ve been told, they’ve forgotten, or I’m meant to tell them and we don’t know which one it is.” [Akal, GP] “The letter says, ‘We were able to visualise L4-T5. Appearance of VFA were suspicious of vertebral fracture.’ I don’t know where that is [laughter]. I know it’s in your back somewhere but I thought, ‘Is it low, medium or high up in the back.’ It would have been nice to have known where it actually was.” [Georgia, patient with vertebral fractures] “Oh God, it was awful [laughter]. I’m sorry, I just find it hysterical. It’s like, ‘Oh my God I’ve broken my back.’ You know, you’ve been given this information of a spinal fracture, that sounded pretty serious to me. And obviously I haven’t broken my back at all, it’s not quite like that.” [Susan, patient with vertebral fractures]
**Planning and scheduling of treatment**		
**Facilitators**	**Barriers**	**Illustrative quotations**
Patients are proactive in arranging appointments with their GP and asking for treatment for their vertebral fractures.	Healthcare professionals forget to prescribe bone protection therapies as they are focused on the immediate injury. Healthcare professionals in primary care are unsure whether treatment has been initiated by healthcare professionals at the hospital. Lack of clarity over referral criteria to specialist services in hospital for management.	“So [prescription of bone protection therapies] could quite easily get overlooked there and you know you might look at it and think, ‘oh well I’ll check if their pain’s okay’, and if their pain’s okay you might not be at the top of your mind thinking ‘oh gosh they do need bone protection’. So I think it could potentially get missed there. [Sophie, GP] “In interface clinics I think they aren’t prescribed bone protection medication, and they have had previous low impact fractures” [Ava, FCP] “There are cases where a patient’s been admitted to hospital. Been in for a long time or been in for something else. Picked up some fractures and then we’re at the stage where we don’t know if they’ve been told… We’re supposed to deal with it and make the referrals and manage things moving forward and we don’t know what’s being told. Sometime later you find out that they didn’t know and that hasn’t been managed or it’s been forgotten about.” [Akal, GP]

### Phase 2: translation of findings to develop recommendations

#### Participant characteristics

A total of 18 participants took part in three meetings. Two online meetings included eight participants (Groups 1 and 2) and one telephone meeting (for participants without access to the internet) included two participants (Group 3). The two online meetings took between 1 h, 46 min and 2 h. The telephone meeting took 1 h 39 min. Participant characteristics are displayed in Tables 4 and 5. All names are pseudonyms.

**Table 4:** Participants in the stakeholder group meetings: healthcare professionals

Group number	Pseudonym	Age range	Sex (M/F)	PT/FT	Estimated number of fractures diagnosed per year	Role
1	Caitlin	50–54	F	PT	10–12	Advanced Clinical Practitioner
1	Emily	45–49	F	FT	N/A	Fracture Liaison Specialist Nurse
2	Josie	50–54	F	FT	Varies	Advanced Practitioner/DXA Service Lead
1	Amelie	45–49	F	FT	4	Health Education England/National Institute for Health Research Integrated Clinical and Practitioner Academic Clinical Lecturer in Physiotherapy
2	Ivy	45–49	F	PT	10–15	Consultant Geriatrician
2	Elena	50–54	F	PT	2	General Practitioner and Musculoskeletal doctor

Acronyms: F = female, M = male, NB = non-binary, X = other

**Table 5:** Participants in the stakeholder group meetings: women with vertebral fractures and family members of people with vertebral fractures

Group number	Pseudonym	Age range	Sex (M/F)	Ethnicity	Year first diagnosed	Number of vertebral fractures	Role
1	Wendy	60–64	F	Mixed	2003	11 or 12	Patient
2	Naomi	75–79	F	White	2019	5	Patient
2	Delilah	60–64	F	White	2019	7	Patient
1	Ruby	60–64	F	White	2011	1	Patient
2	Autumn	75–79	F	White	2018	5	Patient
2	Bella	65–69	F	White	1984	13	Patient
1	Sadie	70–74	F	White	2000	Not applicable	Carer
1	Caroline	70–74	F	White	2005	2	Patient
1	Jade	60–64	F	White	2020	5	Patient
2	Eden	60–64	F	White	2016	30	Patient
3	Reagan	85–89	F	White	Unsure	1	Patient
3	Daisy	70–74	F	White	2015	Unsure	Patient

Acronyms: F = female, M = male

### Findings

Overall, the three groups suggested a total of 64 recommendations. The maximum score was ‘3’ for each item (3 = very important, 2 = important, 1 = not important). The total sum of all the scores was calculated, and divided by the number of people in each group, to give an average score for each item. Of the 64 recommendations, 32 met the required average score (range: 1.75-3).

Recommendations with an average score of > 2.5–3 were included in the top 10–12 recommendations identified in each meeting. Following synthesis, 14 recommendations were prioritised and collated across all three groups [43, 44]. Recommendations were divided into those for healthcare professionals in primary care (*n* = 9) and patients (*n* = 5). See Table 6 for our final list of 14 recommendations.

**Table 6:** Final recommendations to improve identification of people with osteoporotic vertebral fractures identified using the Nominal Group Technique

**Patient appraisal, self-management and decision to consult healthcare professional**	
**Recommendation**	**Key group**
1. Information for patients about what vertebral fractures are and the symptoms, such as such as severe pain that doesn’t improve with time and curvature of the spine.	Patients
2. Information for patients about the risk factors for vertebral fractures, such as the menopause and steroids.	Patients
3. Encourage patients to consult their GP if they have symptoms that suggest they may had had a vertebral fracture, particularly (although not always) after a fall or injury.	Patients
**Healthcare professional appraisal, investigations, referrals and appointments**	
4. Guidance for healthcare professionals in primary care about the risk factors for vertebral fractures when patients present with back pain, such as menopause and steroids.	Healthcare professionals
5. Guidance for healthcare professionals that people who do not have symptoms may have vertebral fractures. Information that other groups that are not ‘typical’ osteoporotic patients such as younger women and men may also be at risk.	Healthcare professionals
6. Guidance for healthcare professionals in primary care about the symptoms of vertebral fractures, such as severe pain that doesn’t improve with time and curvature of the spine.	Healthcare professionals
7. Guidance for GPs on how to carry out a full and comprehensive assessment for vertebral fractures, including physical examination.	Healthcare professionals
8. An evidence-based tool for healthcare professionals in primary or secondary care, outlining who needs to be referred for imaging for vertebral fractures.	Healthcare professionals
9. Guidance for healthcare professionals about referral pathways for vertebral fractures, such as how to refer for imaging and who is responsible for prescribing bone protection therapies.	Healthcare professionals
**Communication of diagnosis**	
10. Communicate the diagnosis to patients verbally (face to face if possible) followed by a patient-friendly letter. Explain to patients what vertebral fractures are, how they are treated, and the implications of having a vertebral fracture.	Healthcare professionals
11. Use clear and consistent terminology to describe vertebral fractures.	Healthcare professionals
12. Direct patients to patient-friendly information about vertebral fractures and make these available in GP surgeries.	Healthcare professionals
**Planning and scheduling of treatment**	
13. Information for patients about the risks of having further fractures, the importance of bone health and bone protection therapies.	Patients
14. Encourage patients to ask healthcare professionals about bone protection therapies if they are not offered. If they have fractured while on bone protection therapies, encourage patients to ask for a review of their medication.	Patients
9. Guidance for healthcare professionals about referral pathways for vertebral fractures, such as how to refer for imaging and who is responsible for prescribing bone protection therapies (*Recommendation as above*)	Healthcare professionals

### Phase 3: co-producing resources

#### Participant characteristics

A total of 12 participants took part in two co-production workshops (Tables 7 and 8). These included five healthcare professionals, six patients and a representative from a patient advocacy group. Patients were aged between 64 and 76 years (average 70 years). All names are pseudonyms.

**Table 7:** Participants in the co-production workshops: healthcare professionals

Pseudonym	Group	Age range	Sex (M/F)	Part time/full time	Years since qualifying	Years in current role	VFs per year	Role
Jasmine	1, 2	40–44	F	PT	20	8	N/A	Specialist Rheumatology Nurse
Faith	1	45–49	F	PT	25	3 months	4	First Contact Physiotherapist
Ava	2	55–59	F	FT	31	2	2	First Contact Physiotherapist
Abigail	2	35–39	F	PT	18	15	100+	Physiotherapist
Chloe	2	40–44	F	PT	14	14 months	N/A	First Contact Physiotherapist

Acronyms: F = female, M = male

**Table 8:** Participants in the co-production workshops: men and women with vertebral fractures and carers of people with vertebral fractures

Pseudonym	Workshop	Age range	Sex (M/F)	Ethnicity	Number of VFs	Year first diagnosed	Role
Lucina	1	75–79	F	White	Carer	N/A	Carer
Eden	1, 2	60–64	F	White	30	2016	Patient
Summer	1, 2	70–74	F	White	Carer	N/A	Carer
Reese	1, 2	70–74	F	White	3	2018	Patient
Freya	1, 2	75–79	F	White	Unsure	2019	Patient
Valerie	2	65–69	F	White	4	2021	Patient

Acronyms: F = female, M = male

Co-production of knowledge sharing resources was achieved in three steps. See Box 1 for illustrative quotes from co-production workshops.

### Step 1: ideas generation workshop

Three key considerations were discussed in initial idea generation workshops to develop knowledge sharing resources: key groups and messages, resource design and dissemination strategies.

(i) Key groups and messages.

Participants agreed with the need to encourage treatment-seeking and early diagnosis, by making patients and healthcare professionals aware of symptoms and risk factors. They highlighted the need to identify and support key groups in primary care who have an expanding role in identifying vertebral fractures, particularly First Contact Physiotherapists, along with GPs who do not necessarily have a specialist interest in osteoporosis. Participants were keen to appeal to lower risk and diverse groups, who were most at risk of missed diagnosis.

(ii) Resource design.

Participants suggested a range of potential resources. These included a clinic poster that could be displayed as a prompt for first contact physiotherapists to support identification and referral, as well as an online referral pathway that healthcare professionals could click for information about each stage. For patients, participants suggested leaflets and a poster with signs and symptoms to encourage treatment-seeking. Participants expressed a preference for infographics, as there was concern that stock images might not convey sufficient diversity. There was a desire that images reflected “seriousness” and avoided “ageist” images of older women.

(iii) Dissemination strategies.

Participants identified a range of targeted dissemination strategies. This included trusted professional organisations, existing NHS platforms, and disseminating patient resources in public spaces to reach those who had not yet entered the healthcare system.

### Step 2: development of prototype resources

**Fig. 2:**
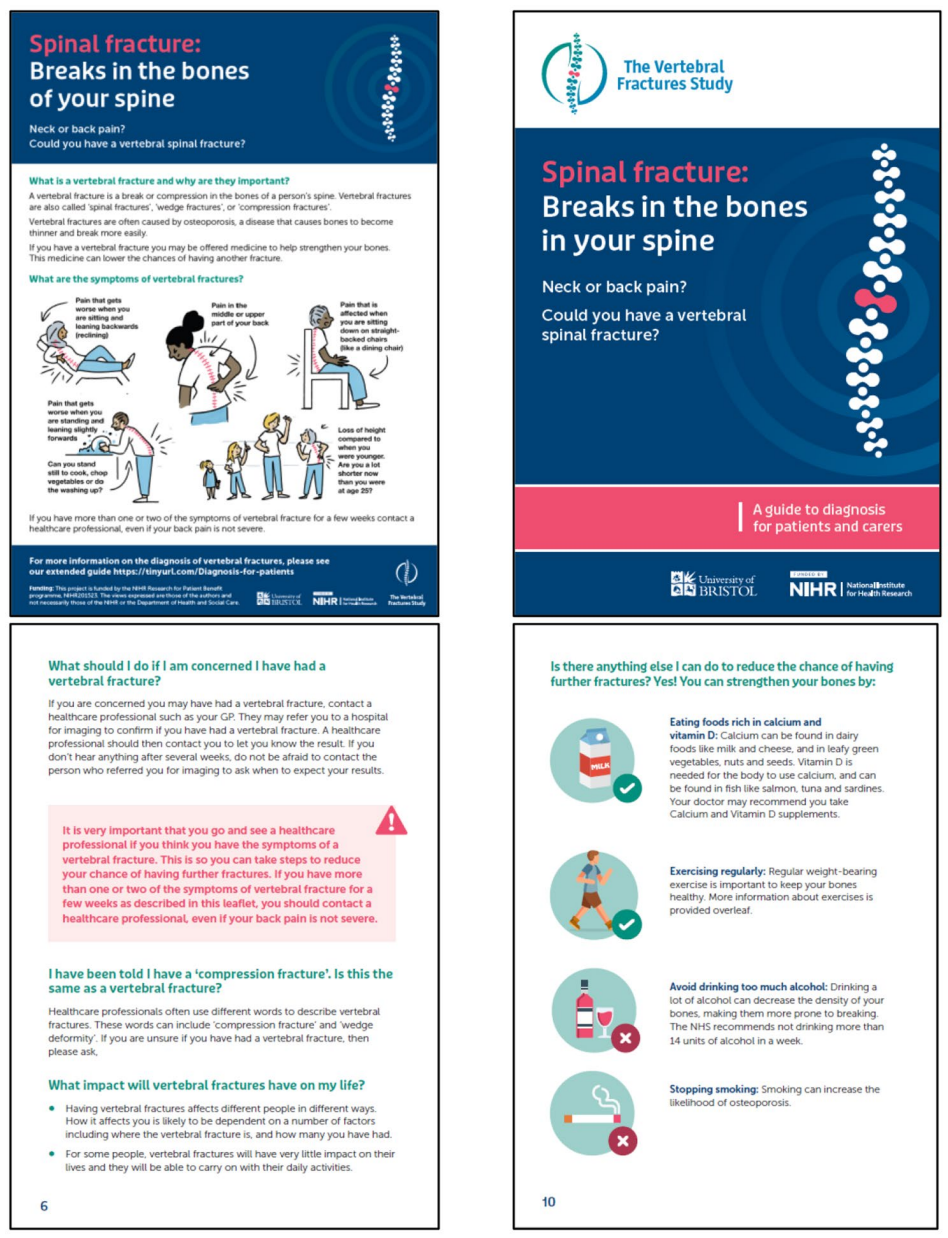
Final designs patient resources

**Fig. 3:**
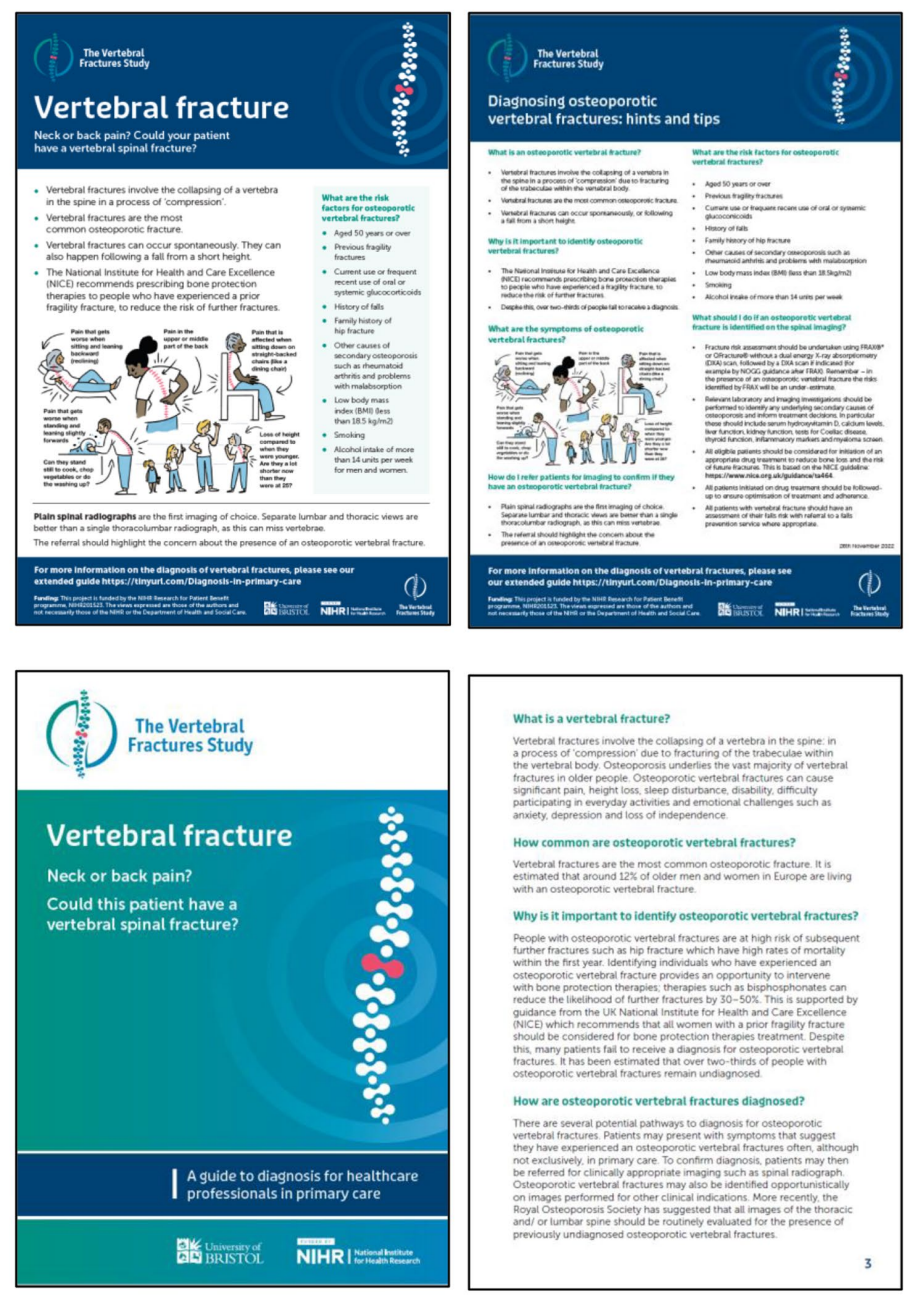
Final designs healthcare professional resources

Following Stage 1, prototype resources were developed: posters, information booklets and a short summary of hints and tips for diagnosis for healthcare professionals (see Figs. 1 and 2 below). An infographic describing symptoms of vertebral fractures was developed as a standalone design (Fig. 3). All images used within the resources were designed by illustrators, or were stock images. No identifying information has been included.

Three key considerations were identified: design, content and readability.

The suite of resources was based on suggestions made by the community of practice. On reflection, the team decided it would not be feasible to develop an online referral pathway given national variation in care pathways [45]. Consideration was given to ensure resources appealed to diverse populations; the infographic included men and women and a range of ethnicities and ages. Information was provided in a question and answer structure. Content was informed by guidance from NICE [46] and the National Osteoporosis Guidelines Group (NOGG) [47] to ensure it reflected best practice. Symptoms of vertebral fractures included in the infographic were based on the VFrac checklist, an evidence-based tool to identify descriptions of back pain in patients presenting with vertebral fractures [48]. To enhance readability, patient resources were reviewed and refined by patient involvement representatives.

### Step 3: Consultation workshop

Resources were evaluated in relation to their acceptability, adequacy, tone and readability.

(i) Acceptability.

All were enthusiastic about the resources. Patients felt that they accurately reflected their own symptoms and experiences and that resources would provide a prompt for treatment-seeking. Participants felt healthcare professionals’ resources were accessible to those who did not have a specialist interest. Visual reminders on posters were seen as an effective way of prompting healthcare professionals to carry out further investigations.

(ii) Adequacy.

Participants felt the resources gave them new knowledge and helpful advice. Several changes were suggested, including changes to the wording to discourage patients with generalised back pain from requesting unnecessary imaging and encouraging patients to be proactive in contacting their GPs if they felt they had symptoms. Healthcare professionals requested further information about potential mechanisms of injury and signposting to guidelines that informed the resources to enhance credibility.

(iii) Tone.

Participants were satisfied with the tone of resources. They suggested adding a description about the purpose of the booklet to provide clarity for readers and a “snappy” title. To engage patients, many felt it was important to acknowledge the impact of vertebral fractures on their lives. Further changes included amending the order of risk factors and recommendations to improve bone health, to remove any potential stigma and blame by placing “stopping smoking” and “reducing alcohol intake” at the bottom. Participants agreed the infographic was “friendly”, “engaging” and accessible to those with difficulty reading. They valued the diversity of images.

(iv) Readability.

The information in the resources was generally considered to be easy to understand, accessible and an appropriate length. Healthcare professionals highlighted the need to present information in the order of the care pathway to improve “flow”. Participants suggested explaining that “vertebral fractures” and “spinal fractures” are the same thing.

Changes were made to the information resources in consultation with an osteoporosis patient support group. Patient resources were translated into Punjabi, Urdu and Somali to enhance inclusivity.

**Fig. 4:**
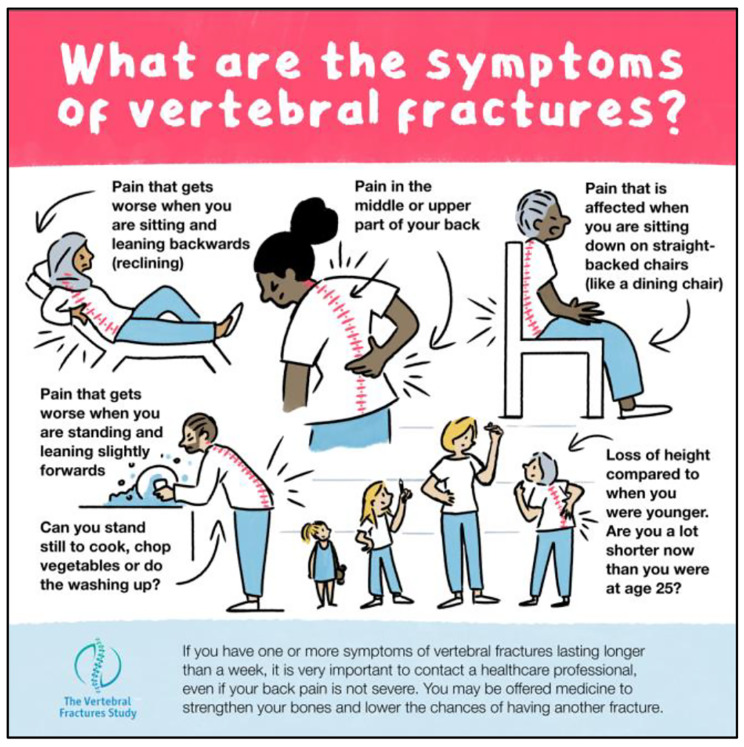
Final design infographic

Examples of the resources are provided in Figs. 2, 3 and 4 below. Copies of the resources are available in Additional Files 2–6.

## Box 1: illustrative quotations from co-production workshops

### Step 1: idea generation workshop

*“You could have a picture of a spine with sort of like a red compressed fracture or whatever you want to call it at the moment and say, ‘If you have severe back pain it could be this, it could be a vertebral fracture. See your doctor for advice.’”* [Ava, FCP].

*“Maybe [having] a poster checklist or something like that [would be helpful]. Something easy you can refer to in a time-pressured clinic.”* [Jasmine, Specialist Rheumatology Nurse].

*“I feel that moving forward a lot of medical leaflets would be better if they had the graphics, the emphasis on the words and the graphics rather than images of a certain part of the population.”* [Freya, 76, patient with vertebral fractures].

*“When you’re wanting to promote the cover maybe you need something which tells you that it’s a vertebral fracture study rather than a tea party… My life has been destroyed by it and we cannot have it as a light-hearted thing.”* [Eden, 64, patient with vertebral fractures].

### Step 3: knowledge mobilisation workshop

*“Personally I think [the patient resources] would get people thinking. I think that a lot of people – and I include myself – don’t really want to always face up to problems. But you’ve got a problem and it’s nagging away.”* [Eden, 64, patient with vertebral fractures].

*“So if you’ve got ongoing spinal pain with these things and then* [it says in the resource], *‘Could it be a fracture in my spine? It’s okay to ask.’* [Patients should be clear] *it’s okay to ask your GP”.* [Abigail, Physiotherapist]

*I wondered* [if] *how they happen is missing. Because often we diagnose them in GP practice because the person has turned the mattress or something or they’ve bent down to pull up weeds. So it can happen for these kind of innocuous reasons and then we’ve obviously got the warning signs.*

[Ava, First Contact Physiotherapist]

*“I did wonder just a bit more broadly whether you could say something on the front that says what’s in it. Because obviously the focus for you is identifying or getting a fracture diagnosed to reduce the chance of another fracture. It’s not a leaflet that’s all about pain management.”* [Representative, Osteoporosis patient support group].

*“I think we’re in an era of encouraging self-management of chronic conditions and therefore I really welcome this… I’ve been struggling for the last six months actually to know about what I can do to manage my condition and therefore I think it’s really important to include this.”* [Valerie, 65, patient with vertebral fractures].

## Discussion

The coproduced knowledge sharing resources provide accessible information to healthcare professionals in primary care and patients to improve care pathways for vertebral fractures. To our knowledge, they are the first resources that are specifically designed to aid identification and treatment by encouraging treatment-seeking for patients who may not have entered the healthcare system and improve recognition of symptoms and risk factors amongst healthcare professionals and provide management guidance without a specialist interest.

Resources developed in partnership and through co-creation can support health literacy in which people have knowledge of their health, illness and forms of management. The right information can improve health outcomes. For example, by being provided with readable, high-quality information, patients have the opportunity to be empowered by patient education resources, being able to then make informed decisions about their treatment [49].

Health literacy is important for older adults and people with long-term conditions, with lower levels of health literacy associated with a lower quality of life [50]. However, there are limitations to providing patient information. A review of patient information materials in primary care found that only around a quarter (24.3%) met the reading level that the material had been designed for, and therefore a considerable amount of text-based health information is inaccessible to many patients [51]. For this reason, the use of plain language in patient-centred materials is now recommended to support knowledge [52]. Simplified language has been shown to be particularly effective when accompanied by images, which have been shown to improve knowledge, understanding and recall of information [53]. The inclusion of pictures in addition to text, such as the explanatory images showing the symptoms of osteoporotic vertebral fractures in our infographic, have been shown to improve understanding [54, 55].

This study used patient-identified and co-created resources to improve patient education and awareness of osteoporotic vertebral fractures. Posters and information leaflets have also been successfully used in reaching a target audience of adults over 65 with depression, using posters and leaflets displayed in the waiting rooms of GP surgeries, and handed out at ’flu clinics [56]. In a recent systematic review, a combination of posters, leaflets and other media in community settings were found to be the most effective [57]. For example, leaflets were found to be more effective than films in patient education for Lyme Disease [58].

How information is provided is as important as the content. Older adults may prefer written education materials in hard copy, rather than digital version [59]. In a recent systematic review, a combination of posters, leaflets and other media in community settings were found to be the most effective at reaching older people from the population [57]. For instance, in research focused on provision of information to older people about depression, posters and leaflets displayed in GPs’ waiting and handed out at ‘flu clinics were found to reach those for whom the information was intended [56]. Although the effects of patient education in osteoporosis have been explored, these have been inconclusive, due to a need for further research in this area [60]. More effective, targeted patient information has the potential to improve health outcomes, by contributing to patient knowledge, engagement and satisfaction and wider improvements in health [51, 61].

In the longer term, the resources developed in the study aim to support diagnosis though increasing awareness of the signs and symptoms of osteoporotic vertebral fractures. This work supports national initiatives to improve pathways to diagnosis such as the Royal Osteoporosis Guidance for the management of symptomatic vertebral fragility fractures [62]. Although it can be argued that there are a lack of incentives for healthcare professionals to monitor osteoporosis in primary care, this research complements the VFrac study, which aims to produce and evaluate an easy-to-use checklist for primary care professionals to ascertain whether patients presenting with lower back pain should be referred for spinal radiographs to diagnose vertebral fractures [48]. Osteoporotic vertebral fractures have been estimated to cost the NHS over £4 billion each year [63], therefore improvement to diagnosis of fractures provides an opportunity for the NHS to make considerable cost savings.

To achieve maximum impact, we are liaising with academic, clinical and charity networks to support national dissemination and promotion. Resources have been published through a range of relevant professional bodies and community organisations. To enhance inclusivity, patient resources have been translated into Punjabi, Urdu and Somali and are being disseminated through Caafi Health (www.caafihealth.com) a grassroots organisation in Bristol, North Somerset and South Gloucestershire that provides accessible health information, support and education. This includes online webinars for patients and providers in the Bristol Inner City Primary Care Network.

### Strengths and limitations

Care was taken to elicit and value input from healthcare professionals and patients for each of their own co-created resources. In research involving professionals and patients it is important to be mindful of the potential for power imbalance, which is acknowledged in work on Communities of Practice (COP) [64]. We were guided by Swaithes and colleagues (2023), whose research also included a range of views in a COP that included healthcare professionals, commissioner, academic, patient and members of the public [65]. In our study we supported participants to access the remotely-delivered CoP workshops, for instance providing technical help if needed. We provided everyone with clear descriptions of their roles and responsibilities in each session, and we gave plain language definitions and explanations of terminology and care pathways associated with osteoporotic vertebral fractures. We hoped that this helped patients, their carers and family members, and healthcare professionals to contribute as fully as possible to co-produced decisions and final resources [65].

All interviews were undertaken by a female researcher (SEB) based in a University and with background in research with vulnerable populations. Interviewees all seemed willing to share their experiences although it is always important to acknowledge that we may not know the extent or presence of any impact of researcher’s identity and background on data collection.

We decided to build on the Model of Pathways to Treatment [21] to provide a comprehensive understanding of the cognitive, behavioural or organisational factors that contribute to delays to diagnosis. The Model is designed to explore patients’ views and therefore enables understanding of, and experiences of, the care pathway. The Model provided us with a theoretically-informed understanding of barriers and facilitators to diagnosis that enabled us to effectively identify targets for change. Based on analysis, we added an additional stage to the Model of Pathways to Treatment – ‘communication of diagnosis’, since this was identified as being a key element of the process that patients encountered and that impacted on time to treatment.

Overall, the co-creation process was well-received. The need to hold co-creation workshops remotely was a unique feature of the pandemic, and was particularly relevant as many of our participants spoke of being clinically vulnerable, and would therefore be discouraged from meeting a large number of others in a face-to-face workshop. Few other researchers have explored co-creation remotely. Thorsen and colleagues (2023) used videoconferencing to co-create assistive devices in partnership with people who have cerebral palsy [66]. Like our study, participants and facilitators could collectively edit the final product, in this case a computer-assisted design (CAD) model, in order to create a more user-centred spoon design. However, this does require a degree of confidence in the use of computer software, and therein lies the disadvantage in remote co-creation. Although our participants had the option during Stage 2 workshops of taking part using their phone on a conference call, in order to better include those who did not have access to the internet. However, for later stages of the research, due to the need for participants to engage with visual materials in real-time, such adjustment was not possible for co-creation workshops. This may therefore have excluded groups with lower levels of digital literacy. We are keen to see how participants without internet access will continue to be represented in the shift towards videoconferencing-based co-creation.

Data collection was carried out during the COVID-19 pandemic, which impacted on care pathways in primary care. To mitigate this, healthcare professionals were asked to recall their experiences pre-pandemic, although asking them to remember a period some time before the study might have impacted data quality. Conducting the study during this period meant that data collection had to be conducted remotely.

Although included in the earlier interviews, we were only able to include one GP in the latter workshop stages of this research. While insight from primary care was also gained from FCPs, future research in collaboration with a greater number of GPs could gain greater feedback and insight.

Despite adoption of strategies to include men and diverse minority ethnic groups, most patients who took part self-identified as women, and all but one were of white ethnicity. Findings and resources may not therefore reflect experiences of other population groups. To address this, we are working with Caafi Health who have provided advice on the applicability of resources to diverse groups. Also, due to the virtual nature of the research, future work involving participants from a wider range of regions could provide greater detail regarding differing socioeconomic, regional and cultural backgrounds.

Additionally, by including those with a confirmed diagnosis of osteoporotic vertebral fractures we were able to include a wide range of experiences of pathways to their diagnosis. However, ethical practice meant we did not include people who did not have diagnosis so that the study did not cause harm through distress. Therefore, there is a chance that the barriers and facilitators relating to treatment-seeking and symptom interpretation may not have been fully explored, particularly in relation to people who do not have awareness of their diagnosis, for whatever reason. Future work could work with patients who do not have knowledge of a vertebral fracture diagnosis, which we suggest could be a stand-alone study. A further potential limitation is that healthcare professionals who contacted the study team to participate were more likely to have an interest in osteoporosis. Nevertheless, we collected a range of experiences and views from those who took part.

User involvement throughout the research and design process, including through the use of co-production methods, conferred several benefits. Incorporating the lived experiences of healthcare professionals and patients has enabled us to develop resources that addressed their real priorities and needs [67]. We have also found that their involvement has helped to facilitate the implementation of resources into practice, since participants are using their own networks and influence to promote their use.

### Future research

Wide differences exist in the identification and treatment of osteoporosis worldwide [68]. Even within the UK, rates of osteoporotic fracture vary by region [69] and levels of socioeconomic deprivation. This may relate in part to presence of risk factors for osteoporotic fractures, such as smoking or alcohol consumption [69–71]. To improve equity of outcomes for all members of the population, future work and service provision may need to focus on provision of support, including resources, for members of communities with higher levels of deprivation. To support wider relevance and uptake of the translated resources, further work could evaluate their usability and underpin further development of translations for other language groups.

Further work is needed to understand the impact of these resources on the identification and management of vertebral fractures. Such work would help to refine resources and modify implementation strategies to ensure maximum engagement and impact. Pharmacological management and starting treatment as is the last stage in the Model of Pathways to Treatment [21]. Future work exploring information needs relating to later stages in patients’ diagnostic and treatment journeys could be beneficial, as patients may not always receive timely diagnosis and treatment of their osteoporotic vertebral fractures. Additionally, the cognitive behavioural and organisational factors that help or hinder diagnosis and treatment identified in this study, along with stakeholder group recommendations, may provide the basis for the development of future interventions to support diagnosis and treatment.

For example, it is not always clear which clinical features should provide the prompt for a referral for radiographs in a person with possible osteoporotic vertebral fractures Similarly, healthcare professionals in this study identified that posters displayed in clinics could provide prompts for first contact physiotherapists to consider identification and referral. The widely used FRAX tool estimates the potential probability of a hip or osteoporotic fracture [72] but the Vfrac tool offers a novel ability to identify which patients with back pain should be offered spinal radiographs. Vfrac uses 15 questions that can be asked by a practice nurse and are based on descriptors that reflect pain experiences of people with osteoporotic vertebral fracture [48]. To maintain consistency and to reflect previous findings about pain experiences, we used Vfrac symptom descriptors to underpin co-creation of the patient infographic.

Healthcare professionals who took part in the study indicated that there are international and regional differences in the treatment of osteoporotic vertebral fractures. In the UK, patients are assessed either within a dedicated fracture liaison service (FLS) on in an osteoporosis clinic, if available. The approach varies according to local care pathways. Furthermore, although multidisciplinary FLS services were recommended in recent clinical guidelines for osteoporosis [73], a recent Scorecard for Osteoporosis in Europe (SCOPE) 2021 report indicates that only around 50% of hospitals in the UK have a FLS [74].

## Conclusions

This study used qualitative methods to develop knowledge sharing resources for patients and healthcare professionals in primary care to aid in the identification and management of vertebral fractures. Dissemination of knowledge sharing resources to a range of stakeholders provides the potential for substantial reach and spread. Further work is now needed to understand the impact of these resources on the identification and management of vertebral fractures.

### Electronic supplementary material

Below is the link to the electronic supplementary material.

Additional file 1

Additional file 2

Additional file 3

Additional file 4

Additional file 5

Additional file 6

## Data Availability

Anonymised data from interviews and co-production workshops will be accessed via the University of Bristol Research Data Repository. Access to the data will be made available to researchers for ethically approved research projects, following a six-year embargo period, which ends on 13th December 2028. Data can be accessed on the understanding that confidentiality will be maintained and after a Data Access Agreement has been signed by an institutional signatory. No authentic request for access will be refused. Data will be available from the 13th December 2028 at the University of Bristol data repository, data.bris, at 10.5523/bris.3j41hce5foycn24m7zql4zgr9t. The data custodian is Dr Sarah Drew (sarah.drew@bristol.ac.uk).

## Declarations

### Authors’ information (optional)

SB is a chartered psychologist with an interest in the self-management of long-term musculoskeletal conditions.

RGH has a background in social anthropology and interdisciplinary research expertise in long-term health conditions.

NW is an academic physiotherapist with expertise in musculoskeletal research and implementation of best practice.

SD is a sociologist with an interest in applied health research for musculoskeletal conditions.

## Abbreviations

CoP: Community of Practice
FCP: First Contact Physiotherapist
GP: General Practitioner
