# Supplementary material for: Improving patients’ experiences of diagnosis and treatment of vertebral fracture: co-production of knowledge sharing resources
Source: BMC Musculoskelet Disord. 2024 Feb 21;25:165. doi: 10.1186/s12891-024-07281-9 (PMC10880218; doi:10.1186/s12891-024-07281-9)
Supplement: Supplementary file 1 — Additional file 1 [file 12891_2024_7281_MOESM1_ESM.pdf]

Additional File 1: The study response pack and taxonomy provided to participants in advance of the Stage 2 meeting.

FUNDED BY

NIHR | National Institute for Health Research

University of BRISTOL

Understanding and improving patient experiences of diagnosis for vertebral fracture: a qualitative study

STUDY RESPONSE PACK

You have kindly offered to take part in a Zoom meeting for this study which will be held on:

- **[Date and time]**

The aim of our meetings is to develop 10 recommendations to improve the diagnosis of vertebral fractures.

What is the purpose of this study?

- Breaking a bone in the spine (vertebral fracture) is very common in people over fifty. Identifying vertebral fractures means healthcare professionals can prescribe bone protection therapies that can reduce the risk of further breakages. However, many people go undiagnosed.

- We have already talked to a range of patients and healthcare professionals in primary care to understand their experiences of identifying vertebral fractures and starting treatment. They have highlighted a number of issues that make this more difficult (barriers) or easier (facilitators).

- In our meetings we will use the information we have collected to help develop recommendations to make diagnosing vertebral fractures and starting treatment quicker and easier.

- This information will be used to help improve diagnosis and management. It will do this by helping us to develop new information resources for healthcare professionals and patients to help identify vertebral fractures and start treatment more easily. These resources will be widely and freely available.

What will happen <u>before</u> our first meeting?

Before the first meeting we sent you this information pack so you can find out more about what taking part will involve and familiarise yourself with the barriers and facilitators to diagnosis and starting treatment that we have already identified.

- In this pack you have been provided with the following information:
    - An overview of the task
    - The results from previous work that we have carried out to identify barriers and facilitators to the diagnosis of vertebral fractures.

- Please read this pack to help you understand what taking part will involve and the barriers and facilitators that we have already identified.

What will happen in our meeting on [Meeting date]?

The purpose of this meeting will be to generate recommendations to improve the diagnosis and treatment of vertebral fractures based on the barriers and facilitators that we have already identified.

- **Introduction to the study:** Sarah will remind you of the purpose of the meeting, inform you how we will develop recommendations and provide you with an overview of the barriers and facilitators that we have identified in our previous work.

- **Part 1: Silent generation of ideas**: Based on the barriers and facilitators that we have identified, you will be asked to write down ideas for recommendations to improve the diagnosis of vertebral fractures.

Your ideas for recommendations.
1. My idea for a recommendation is... ________
____
____

We have included several sheets for you to write down your recommendations at the end of this document. You may also use your own notepad. Please have a pen handy!

- **Part 2: Presentation of recommendations**: You will take turns to share your ideas or recommendations with the group. Sarah will write down these recommendations as you share them.

- **Part 3: Refinement of ideas:** We will discuss the meanings of these recommendations and why they are important as a group.

- **Part 4: Rating:** Your task will be to rate each of these recommendations using a scale:

1 = Not important, **2** = Important, **3** = Very important

An example of this rating is below:

Recommendation 1: Better ice cream flavours on offer for patients

- 1 = Not important
- 2 = Important
- 3 = Very important

- Sarah will sum everyone’s scores. The scores will be totaled and the top 10 recommendations identified.

- **Part 5: Discussion:** We will discuss and reflect on the top 10 recommendations identified as a group.

- **Part 6: Re-rating:** Your task will then be to re-rate each of the recommendations using a scale, as before:

  1 = Not important, **2** = Important, **3** = Very important

- Sarah will sum everyone’s scores. The scores will be totaled and the final top 10 recommendations identified.

- **Part 7: Final results and group discussion**: We will discuss and reflect on the final top 10 recommendations identified as a group.

How are vertebral fractures diagnosed?

There are three ways that vertebral fractures can be diagnosed:

1. A patient visits their GP practice with symptoms that suggest they might have had a vertebral fracture. They are then referred by their GP or other healthcare professional to a hospital to have a medical image taken such as X-ray, magnetic resonance imaging (MRI scan) or computerized tomography scan (CT scan). A vertebral fracture can then be confirmed.

2. A patient attends their local Accident and Emergency Department, or sees a consultant in hospital. They are then referred to have a medical image taken such as an X-ray, MRI scan or CT scan. A vertebral fracture can then be confirmed.

3. A patient has a medical image taken for another reason, such as an X-ray, MRI scan or CT scan and a vertebral fracture is found unexpectedly. A healthcare professional such as a radiologist reports the vertebral fracture.

Below is a diagram that shows how vertebral fractures can be diagnosed.

How vertebral fractures (VFs) are diagnosed

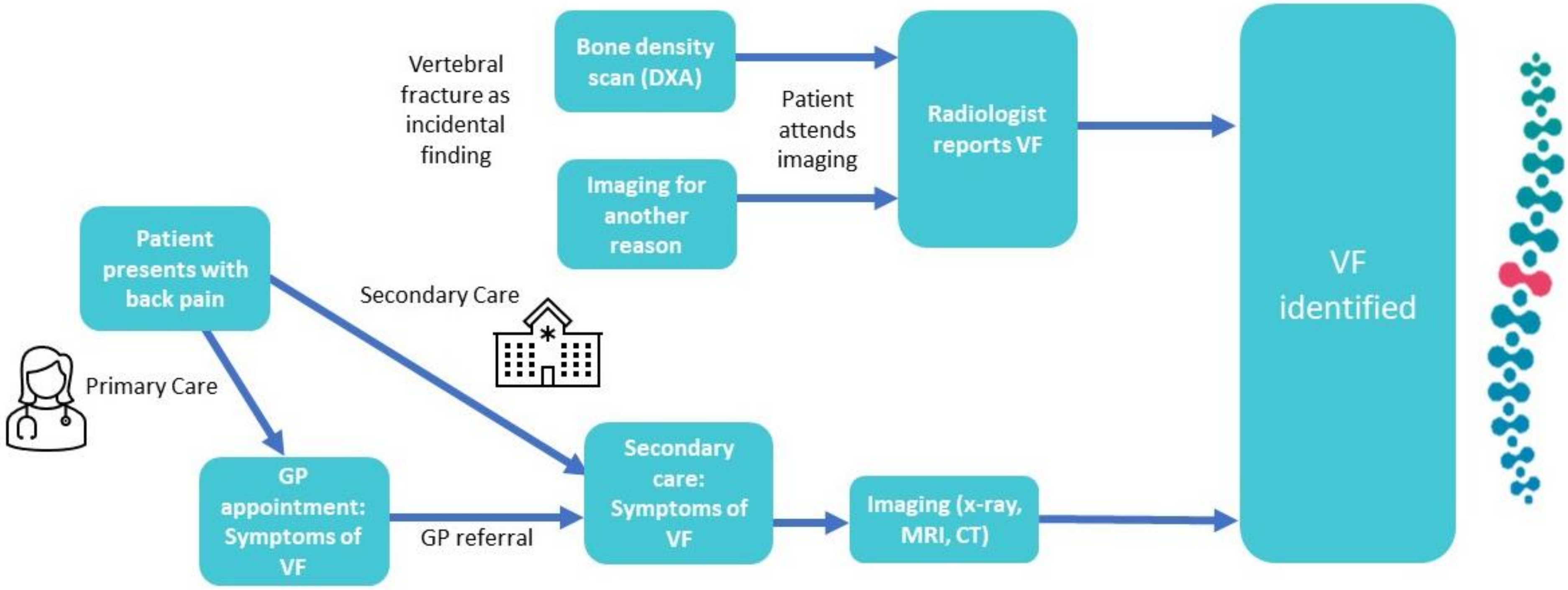

Adapted from: The Royal Osteoporosis Society guidelines for Vertebral Fractures 2018

Barriers and facilitators to diagnosing vertebral fractures

Background

The aim of the first part of this study was to identify barriers and facilitators to the diagnosis of vertebral fractures and starting treatment. These will inform the recommendations that we develop to improve the diagnosis of vertebral fractures in our first meeting.

What we did

We talked to 24 people over the age of 50 who had been diagnosed with a vertebral fracture. We asked them about their experiences of having a vertebral fracture, visiting healthcare services and anything that made it easier or more difficult to get a diagnosis and start treatment.

We talked to 9 healthcare professionals in primary care (general practice), including GPs and physiotherapists with experience of helping to identify people with vertebral fractures. We asked about their experiences of helping to identify patients who may have experienced a vertebral fracture, processes for confirming diagnosis, communicating diagnosis to patients, starting treatment and anything else that makes it easier or more difficult to diagnose patients.

Findings

Below are barriers and facilitators that we identified.

Thinking about and managing symptoms

Facilitators that encourage patients to consult a healthcare professional

- Patients experience pain that is severe and different to any pain they have experienced before.
- Patients' back pain doesn't get better with time.
- Other people notice the symptoms and encourage them to visit a healthcare professional.
- Patients talk to other people about the pain such as friends or family who encourage them to visit a healthcare professional.
- Patients associate their pain with an injury such as a fall.

Barriers that discourage patients from consulting a healthcare professional

- Patients do not know what a vertebral fracture is and what the symptoms are.
- Patients living with osteoporosis are not aware that they are at risk of vertebral fractures.
- Patients do not think the pain is serious.
- Patients mistake the pain for another issue such as a pulled muscle, kidney infection, arthritis or a broken rib.
- Patients have a vertebral fracture that doesn't have any symptoms.
- Patients do not associate their pain with an injury such as a fall.
- Patients choose to manage the pain themselves by taking painkillers or in other ways.
- Patients don't think getting help for their back pain is important and prioritise other conditions or feel that they are too busy.
- Patients feel that they do not want to 'bother' healthcare professionals or 'make a fuss'.

Meeting with healthcare professionals

Facilitators to helping healthcare professionals identify vertebral fractures

- Healthcare professionals in general practice are aware of risk factors, or things that increase the chance of having a vertebral fracture such as age, sex, and low body mass index (BMI).
- Healthcare professionals in general practice have knowledge of the symptoms of vertebral fracture such as height loss and severe back pain.
- Healthcare professionals in general practice can easily refer patients for imaging such as x-rays to confirm diagnosis and to specialists in hospital for assessment.

Barriers that make it more difficult for healthcare professionals to identify vertebral fractures

- Healthcare professionals in general practice and Accident and Emergency (A&E) mistake the symptoms of vertebral fractures for other conditions or causes such as a pulled muscle or broken rib.
- Healthcare professionals in general practice tell patients to wait and see if their vertebral fracture symptoms get better on their own before starting further investigations.
- Patients feel that their healthcare professional does not take their pain seriously and do not want to re-visit them for help if their pain doesn't improve.
- Healthcare professionals find vertebral fractures harder to spot in men and young people because they are less likely to be at risk.

- Healthcare professionals find it more difficult to diagnose vertebral fractures that do not have any obvious symptoms such as pain.
- Healthcare professionals find it hard to get patients with back pain an x-ray in A&E as they have strict criteria on who to image.

Communication of diagnosis

Facilitators to effective communication of diagnosis

- A diagnosis of vertebral fracture is clearly communicated to patients either verbally by their GP or specialist in hospital or through a letter which is patient-friendly and easy to understand.
- Patients are given information about vertebral fractures and osteoporosis when they are diagnosed to help them understand what they are and how to manage them.
- Healthcare professionals explain what a vertebral fracture is to help reduce the feelings of shock and surprise when they are diagnosed.

Barriers to effective communication of diagnosis

- Patients find out about their diagnosis by being copied into medical letters and find some of the ‘big words’ confusing and difficult to understand as they are not explained.
- Healthcare professionals use confusing words to inform patients that they have had a vertebral fracture such as ‘compression fracture’ or ‘wedge deformity’. Patients are therefore unsure if they have had a vertebral fracture.
- Patients find the term “vertebral fractures” alarming as it makes it sound like they have had a catastrophic injury.
- Patients are not clearly told how many vertebral fractures they have had and how they were diagnosed.
- Patients receive contradictory information from healthcare professionals. They are told that their medical image shows they are fine and are then told later by another healthcare professional that they have had a vertebral fracture.
- Healthcare professionals are unclear if a patient has been told about their vertebral fracture as they are being managed by healthcare professionals in hospital and at their GP surgery. Some patients are therefore not informed.

Starting treatment

Facilitators to starting treatment

- Patients are proactive in arranging appointments with their GP and asking for treatment for their vertebral fractures.
- Physiotherapists working at GP practices are able to easily refer patients with vertebral fracture to their GP for treatment.
- Patients are prescribed treatments such as bone protection therapies by healthcare professionals and the importance of these treatments is clearly communicated.

Barriers to starting treatment

- Not all patients who need treatments are prescribed them.
- Healthcare professionals are unsure whether treatment should be started by GPs or healthcare professionals at the hospital.
- Healthcare professionals in primary care need to communicate and work with healthcare professionals in hospital to make sure patients start treatment and find this process difficult and confusing.

We will use these findings to develop recommendations to improve diagnosis, communication of diagnosis to patients and starting treatment.

If you have any questions please contact Dr Sarah Bennett who is a member of the research team. Sarah's contact details are:

Email: [Sarah's email]
Tel: [Study phone number]

We would like to thank you again for agreeing to take part. The research that we do helps to inform better healthcare in the UK. Your participation is vital in this.

Meeting with us on the day: [Date and time]

The meeting will take place on a zoom call from 10am. To join the meeting please copy and paste the link below into your browser.

Meeting link: [Zoom Link]
Meeting ID: [Meeting ID]

Your recommendations

Please use this space to write down your own ideas for recommendations during our Zoom meeting.

Thinking about and managing symptoms: Your ideas for recommendations

1.
2.
3.
4.
5.
6.
7.
8.
9.
10.

Meeting with healthcare professionals: Your ideas for recommendations

1.
2.
3.
4.
5.
6.
7.
8.
9.
10.

Communication of diagnosis: Your ideas for recommendations

1.
2.
3.
4.
5.
6.
7.
8.
9.
10.

Starting treatment: Your ideas for recommendations

1.
2.
3.
4.
5.
6.
7.
8.
9.
10.
