# Supplementary material for: Improving patients’ experiences of diagnosis and treatment of vertebral fracture: co-production of knowledge sharing resources
Source: BMC Musculoskelet Disord. 2024 Feb 21;25:165. doi: 10.1186/s12891-024-07281-9 (PMC10880218; doi:10.1186/s12891-024-07281-9)
Supplement: Supplementary file 2 — Additional file 2 [file 12891_2024_7281_MOESM2_ESM.pdf]

The Vertebral Fractures Study

Vertebral fracture

Neck or back pain?

Could this patient have a vertebral spinal fracture?

A guide to diagnosis for healthcare professionals in primary care

University of Bristol

FUNDED BY NIHR | National Institute for Health Research

The content of this booklet was co-produced by people living with vertebral fractures and their family members and healthcare professionals in primary and secondary care.

Authors

Professor Emma Clark, Professor of Clinical Musculoskeletal Epidemiology and Honorary Consultant Rheumatologist, University of Bristol and North Bristol NHS Trust.

Dr Zoe Paskins, Reader and Honorary Consultant in Rheumatology, Arthritis Research UK Primary Care Centre, Keele University.

Professor Nicola Walsh, Professor of Musculoskeletal Health, University of the West of England.

Professor Rachael Gooberman-Hill, Professor of Health and Anthropology and Director of Elizabeth, Blackwell Institute, University of Bristol.

Dr Sarah Bennett, Senior Research Associate, Bristol Medical School, University of Bristol.

Dr Sarah Drew, Research Fellow, Bristol Medical School, University of Bristol.

Ms Pat Mascord, Patient Representative.

Mrs Yvonne Sadler, Patient Representative.

Advisors

Ms Sarah Leyland, Osteoporosis Specialist Nurse and Clinical Advisor, Royal Osteoporosis Society.

Funding

This project is funded by the NIHR Research for Patient Benefit programme, NIHR201523. The views expressed are those of the authors and not necessarily those of the NIHR or the Department of Health and Social Care.

This work is licensed under the Creative Commons Attribution 4.0 International License. To view a copy of this license, visit http://creativecommons.org/licenses/by/4.0/

25 November 2022

The Vertebral Fractures Study

What is a vertebral fracture?

Vertebral fractures involve the collapsing of a vertebra in the spine: in a process of ‘compression’ due to fracturing of the trabeculae within the vertebral body. Osteoporosis underlies the vast majority of vertebral fractures in older people. Osteoporotic vertebral fractures can cause significant pain, height loss, sleep disturbance, disability, difficulty participating in everyday activities and emotional challenges such as anxiety, depression and loss of independence.

How common are osteoporotic vertebral fractures?

Vertebral fractures are the most common osteoporotic fracture. It is estimated that around 12% of older men and women in Europe are living with an osteoporotic vertebral fracture.

Why is it important to identify osteoporotic vertebral fractures?

People with osteoporotic vertebral fractures are at high risk of subsequent further fractures such as hip fracture which have high rates of mortality within the first year. Identifying individuals who have experienced an osteoporotic vertebral fracture provides an opportunity to intervene with bone protection therapies; therapies such as bisphosphonates can reduce the likelihood of further fractures by 30–50%. This is supported by guidance from the UK National Institute for Health and Care Excellence (NICE) which recommends that all women with a prior fragility fracture should be considered for bone protection therapies treatment. Despite this, many patients fail to receive a diagnosis for osteoporotic vertebral fractures. It has been estimated that over two-thirds of people with osteoporotic vertebral fractures remain undiagnosed.

How are osteoporotic vertebral fractures diagnosed?

There are several potential pathways to diagnosis for osteoporotic vertebral fractures. Patients may present with symptoms that suggest they have experienced an osteoporotic vertebral fractures often, although not exclusively, in primary care. To confirm diagnosis, patients may then be referred for clinically appropriate imaging such as spinal radiograph. Osteoporotic vertebral fractures may also be identified opportunistically on images performed for other clinical indications. More recently, the Royal Osteoporosis Society has suggested that all images of the thoracic and/ or lumbar spine should be routinely evaluated for the presence of previously undiagnosed osteoporotic vertebral fractures.

What are the symptoms of osteoporotic vertebral fractures?

Identification of osteoporotic vertebral fractures is challenging as up to two-thirds of patients do not present with severe symptoms. Where patients do present with symptoms, they are often mistaken for other causes such as muscular pain. Unlike other osteoporotic fractures such as hip fracture, only 20% of osteoporotic vertebral fractures occur following a fall. Many osteoporotic vertebral fractures are caused by everyday activities. However, patients may not be able to identify a specific event or injury.

Symptoms of osteoporotic vertebral fractures can include:

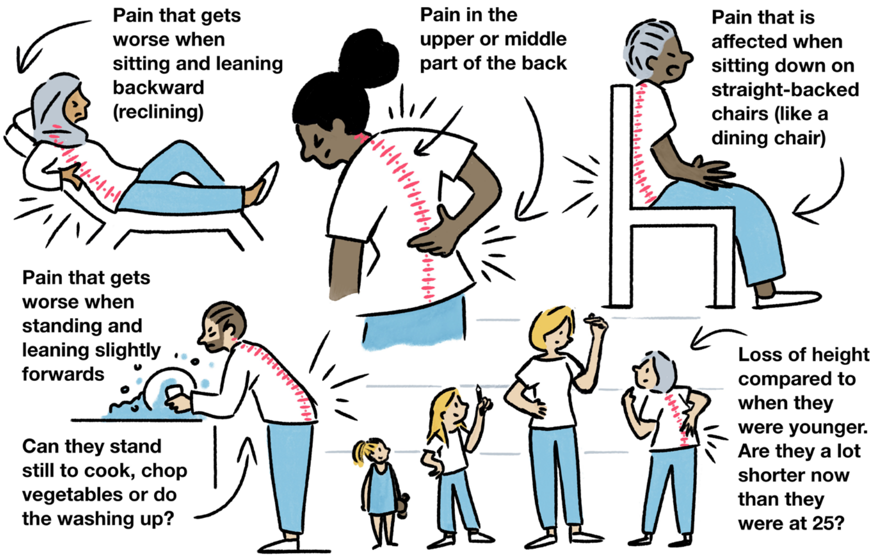

What are common risk factors for osteoporotic vertebral fractures?

- Aged 50 years or over
- Previous fragility fractures
- Current use or frequent recent use of oral or systemic glucocorticoids
- History of falls
- Family history of hip fracture
- Other causes of secondary osteoporosis such as rheumatoid arthritis and problems with malabsorption

- Low body mass index (BMI) (less than 18.5 kg/m2)
- Smoking
- Alcohol intake of more than 14 units per week for men and women

For a full overview of risk factors for osteoporotic fractures see:
National Institute for Health and Care Excellence (NICE) guideline Osteoporosis: assessing the risk of fragility fracture (NICE, 2017a): https://www.nice.org.uk/guidance/cg146

How do I carry out an examination for patients with suspected osteoporotic vertebral fractures?

Healthcare professionals should have a high index of suspicion in patients presenting with back pain, particularly older patients and those with existing risk factors for osteoporosis.

There are no clinical signs which are specific for vertebral fracture.

In the acute phase the spine may be tender to deep palpation and percussion and there may be paravertebral muscle spasm.

Vertebral fractures are associated with a reduction in vertebral height. With increasing number of fractures, height loss and kyphosis may occur.

Where there are concerns about the occurrence of a vertebral fracture(s), recent imaging that includes the spine should be reviewed. If there is none or the symptoms started following previous imaging, patients should be referred for spine imaging.

How do I refer patients for imaging to confirm if they have a vertebral fracture?

Plain spinal radiographs are the first imaging of choice. Separate lumbar and thoracic views are better than a single thoracolumbar radiograph, as this can miss vertebrae.

The referral should highlight the concern about the presence of an osteoporotic vertebral fracture.

What should I do if an osteoporotic vertebral fracture is identified on the spinal imaging?

All patients should have their fracture risk assessed and modified if indicated

- Fracture risk assessment should be undertaken using FRAX®* or QFracture® without a dual energy X-ray absorptiometry (DXA) scan, followed by a DXA scan if indicated (for example by NOGG guidance after FRAX). Remember – in the presence of an osteoporotic vertebral fracture the risks identified by FRAX will be an under-estimate.

- Relevant laboratory and imaging investigations should be performed to identify any underlying secondary causes of osteoporosis and inform treatment decisions. In particular these should include serum hydroxyvitamin D, calcium levels, liver function, kidney function, tests for Coeliac disease, thyroid function, inflammatory markers and myeloma screen.

- All eligible patients should be considered for initiation of an appropriate drug treatment to reduce bone loss and the risk of future fractures.

- This is based on the National Institute for Health and Care Excellence (NICE) guideline Bisphosphonates for treating osteoporosis (NICE, 2017b): https://www.nice.org.uk/guidance/ta464

- All patients initiated on drug treatment to reduce bone loss and risk of future fractures should be followed-up, to ensure optimisation of treatment, compliance and persistence.

Holistic assessment

All patients should have an holistic assessment to determine the impact of the fracture(s) on pain, function and quality of life

» A useful guide from the Royal Osteoporosis Society is available on assessment of symptoms of vertebral fracture including pain, hyperkyphosis, pulmonary symptoms, urinary and gastrointestinal symptoms, psychological symptoms, and falls/falls risk: https://tinyurl.com/vertebralfractures

Treatment should include consideration of pharmacological and non-pharmacological management of pain for everyone.

- All patients with vertebral fracture should have an assessment of their falls risk with referral to a falls prevention service where appropriate

Recommended exercise

All patients should be empowered to undertake exercise to help reduce pain, improve muscle strength and balance, reduce fear of falling, and generally improve quality of life.

The following resources are available on the Royal Osteoporosis Society website:

- For healthcare professionals, a ROS consensus statement 'Strong, Steady and Straight' (ROS, 2019), an Expert Consensus Statement on physical activity and exercise for osteoporosis: https://tinyurl.com/StrongSteadyStraight

- For patients, the following ROS factsheets are available:

  - 'Exercise for back pain after spinal fractures': https://tinyurl.com/BackPainAfterFractures
  - 'Exercises to promote bone and muscle strength': https://tinyurl.com/BoneMuscleStrength
  - 'Exercises to improve balance and muscle strength': https://tinyurl.com/BalanceMuscleStrength
  - 'Exercises for back pain after spinal fractures': https://tinyurl.com/BackPainAfterFracture
  - 'Moving and lifting safely': https://tinyurl.com/MovingAndLiftingSafely

- Healthcare professionals should consider referral to a physiotherapist in the acute setting if the pain is severe and the patient is unable to mobilise and manage daily activities.

All patients with vertebral fracture should receive accurate verbal and written information.

- This should include information about osteoporosis and vertebral fracture to enhance their understanding of the condition, including how medication, nutrition, fall prevention and exercise are important in its management.

- All patients should be informed that having a vertebral fracture may increase their risk of a future fracture (including vertebral and non-vertebral fractures), and that this risk can be substantially reduced by treatment.

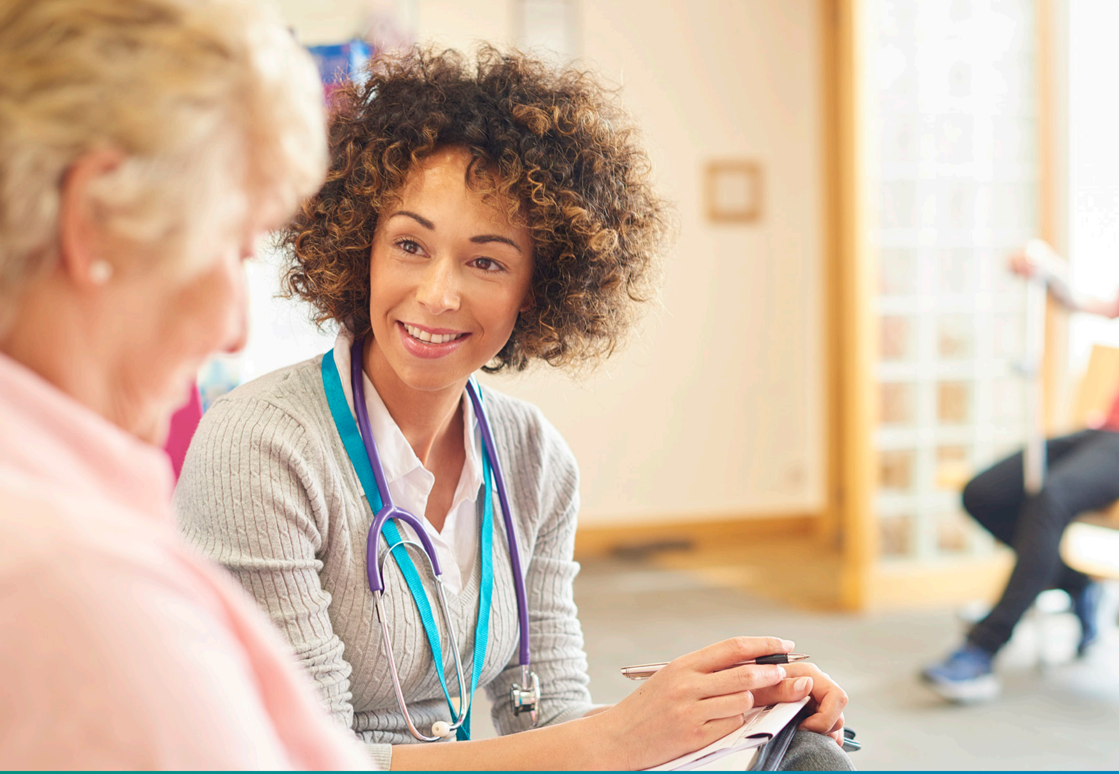

Where else can I find reliable information about osteoporosis and vertebral fractures?

The Royal Osteoporosis Society provides information and advice about osteoporosis and bone health:

www.theros.org.uk

University of BRISTOL

FUNDED BY NIHR | National Institute for Health Research

The Vertebral Fractures Study
