# Supplementary material for: Improving patients’ experiences of diagnosis and treatment of vertebral fracture: co-production of knowledge sharing resources
Source: BMC Musculoskelet Disord. 2024 Feb 21;25:165. doi: 10.1186/s12891-024-07281-9 (PMC10880218; doi:10.1186/s12891-024-07281-9)
Supplement: Supplementary file 3 — Additional file 3 [file 12891_2024_7281_MOESM3_ESM.pdf]

The Vertebral Fractures Study

Vertebral fracture

Neck or back pain? Could your patient have a vertebral spinal fracture?

- Vertebral fractures involve the collapsing of a vertebra in the spine in a process of 'compression'.
- Vertebral fractures are the most common osteoporotic fracture.
- Vertebral fractures can occur spontaneously. They can also happen following a fall from a short height.
- The National Institute for Health and Care Excellence (NICE) recommends prescribing bone protection therapies to people who have experienced a prior fragility fracture, to reduce the risk of further fractures.

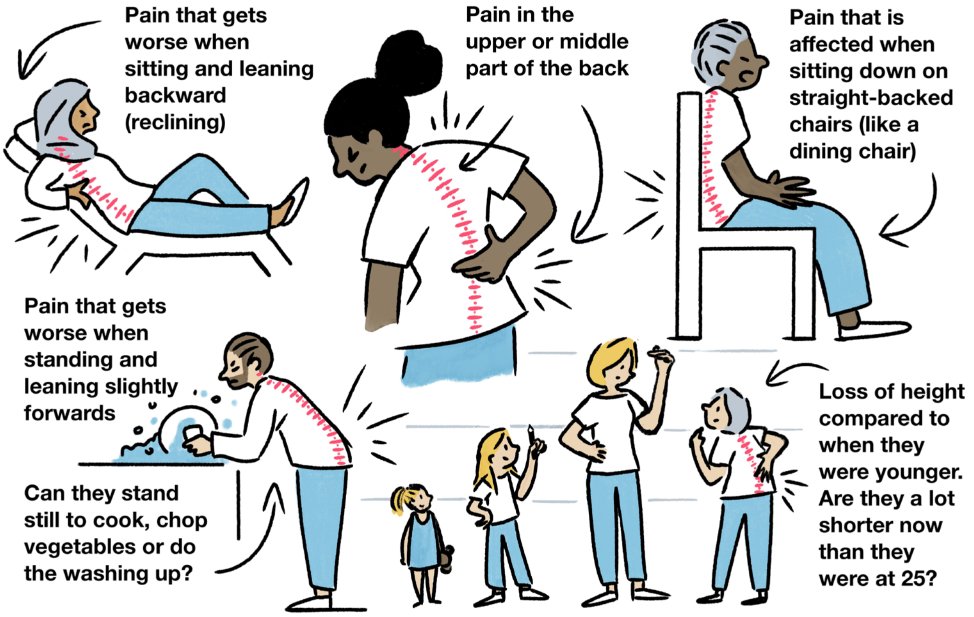

What are the risk factors for osteoporotic vertebral fractures?

- Aged 50 years or over
- Previous fragility fractures
- Current use or frequent recent use of oral or systemic glucocorticoids
- History of falls
- Family history of hip fracture
- Other causes of secondary osteoporosis such as rheumatoid arthritis and problems with malabsorption
- Low body mass index (BMI) (less than 18.5 kg/m2)
- Smoking
- Alcohol intake of more than 14 units per week for men and women.

Plain spinal radiographs are the first imaging of choice. Separate lumbar and thoracic views are better than a single thoracolumbar radiograph, as this can miss vertebrae.

The referral should highlight the concern about the presence of an osteoporotic vertebral fracture.

For more information on the diagnosis of vertebral fractures, please see our extended guide https://tinyurl.com/Diagnosis-in-primary-care

Funding: This project is funded by the NIHR Research for Patient Benefit programme, NIHR201523. The views expressed are those of the authors and not necessarily those of the NIHR or the Department of Health and Social Care.

University of BRISTOL

FUNDED BY NIHR | National Institute for Health Research

The Vertebral Fractures Study
