# Supplementary material for: Improving patients’ experiences of diagnosis and treatment of vertebral fracture: co-production of knowledge sharing resources
Source: BMC Musculoskelet Disord. 2024 Feb 21;25:165. doi: 10.1186/s12891-024-07281-9 (PMC10880218; doi:10.1186/s12891-024-07281-9)
Supplement: Supplementary file 4 — Additional file 4 [file 12891_2024_7281_MOESM4_ESM.pdf]

The Vertebral Fractures Study

Diagnosing osteoporotic vertebral fractures: hints and tips

What is an osteoporotic vertebral fracture?

- Vertebral fractures involve the collapsing of a vertebra in the spine in a process of 'compression' due to fracturing of the trabeculae within the vertebral body.
- Vertebral fractures are the most common osteoporotic fracture.
- Vertebral fractures can occur spontaneously, or following a fall from a short height.

Why is it important to identify osteoporotic vertebral fractures?

- The National Institute for Health and Care Excellence (NICE) recommends prescribing bone protection therapies to people who have experienced a fragility fracture, to reduce the risk of further fractures.
- Despite this, over two-thirds of people fail to receive a diagnosis.

What are the symptoms of osteoporotic vertebral fractures?

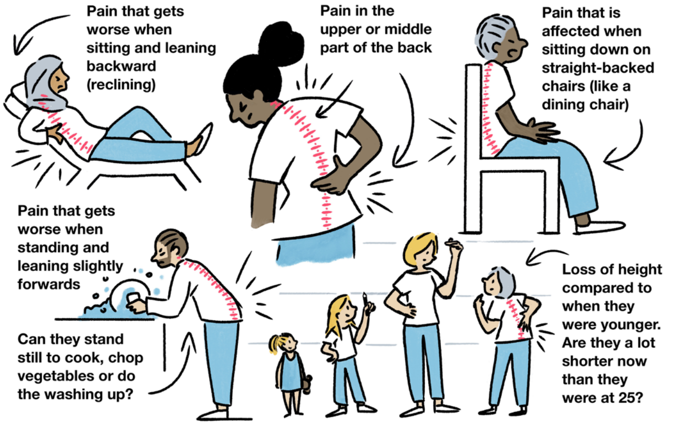

How do I refer patients for imaging to confirm if they have an osteoporotic vertebral fracture?

- Plain spinal radiographs are the first imaging of choice. Separate lumbar and thoracic views are better than a single thoracolumbar radiograph, as this can miss vertebrae.
- The referral should highlight the concern about the presence of an osteoporotic vertebral fracture.

What are the risk factors for osteoporotic vertebral fractures?

- Aged 50 years or over
- Previous fragility fractures
- Current use or frequent recent use of oral or systemic glucocorticoids
- History of falls
- Family history of hip fracture
- Other causes of secondary osteoporosis such as rheumatoid arthritis and problems with malabsorption
- Low body mass index (BMI) (less than 18.5kg/m2)
- Smoking
- Alcohol intake of more than 14 units per week

What should I do if an osteoporotic vertebral fracture is identified on the spinal imaging?

- Fracture risk assessment should be undertaken using FRAX®* or QFracture® without a dual energy X-ray absorptiometry (DXA) scan, followed by a DXA scan if indicated (for example by NOGG guidance after FRAX). Remember – in the presence of an osteoporotic vertebral fracture the risks identified by FRAX will be an under-estimate.
- Relevant laboratory and imaging investigations should be performed to identify any underlying secondary causes of osteoporosis and inform treatment decisions. In particular these should include serum hydroxyvitamin D, calcium levels, liver function, kidney function, tests for Coeliac disease, thyroid function, inflammatory markers and myeloma screen.
- All eligible patients should be considered for initiation of an appropriate drug treatment to reduce bone loss and the risk of future fractures. This is based on the NICE guideline: **https://www.nice.org.uk/guidance/ta464**.
- All patients initiated on drug treatment should be followed-up to ensure optimisation of treatment and adherence.
- All patients with vertebral fracture should have an assessment of their falls risk with referral to a falls prevention service where appropriate.

25th November 2022

For more information on the diagnosis of vertebral fractures, please see our extended guide https://tinyurl.com/Diagnosis-in-primary-care

Funding: This project is funded by the NIHR Research for Patient Benefit programme, NIHR201523. The views expressed are those of the authors and not necessarily those of the NIHR or the Department of Health and Social Care.

University of BRISTOL

FUNDED BY NIHR | National Institute for Health Research

The Vertebral Fractures Study
