# Supplementary material for: Improving patients’ experiences of diagnosis and treatment of vertebral fracture: co-production of knowledge sharing resources
Source: BMC Musculoskelet Disord. 2024 Feb 21;25:165. doi: 10.1186/s12891-024-07281-9 (PMC10880218; doi:10.1186/s12891-024-07281-9)
Supplement: Supplementary file 5 — Additional file 5 [file 12891_2024_7281_MOESM5_ESM.pdf]

The Vertebral Fractures Study

Spinal fracture: Breaks in the bones in your spine

Neck or back pain?

Could you have a vertebral spinal fracture?

A guide to diagnosis for patients and carers

University of Bristol

FUNDED BY NIHR | National Institute for Health Research

The content of this booklet was co-produced by people living with vertebral fractures and their family members and healthcare professionals in primary and secondary care.

Authors

Professor Emma Clark, Professor of Clinical Musculoskeletal Epidemiology and Honorary Consultant Rheumatologist, University of Bristol and North Bristol NHS Trust.

Dr Zoe Paskins, Reader and Honorary Consultant in Rheumatology, Arthritis Research UK Primary Care Centre, Keele University.

Professor Nicola Walsh, Professor of Musculoskeletal Health, University of the West of England.

Professor Rachael Gooberman-Hill, Professor of Health and Anthropology and Director of Elizabeth, Blackwell Institute, University of Bristol.

Dr Sarah Bennett, Senior Research Associate, Bristol Medical School, University of Bristol.

Dr Sarah Drew, Research Fellow, Bristol Medical School, University of Bristol.

Ms Pat Mascord, Patient Representative.

Mrs Yvonne Sadler, Patient Representative.

Advisors

Ms Sarah Leyland, Osteoporosis Specialist Nurse and Clinical Advisor, Royal Osteoporosis Society.

Funding

This project is funded by the NIHR Research for Patient Benefit programme, NIHR201523. The views expressed are those of the authors and not necessarily those of the NIHR or the Department of Health and Social Care.

This work is licensed under the Creative Commons Attribution 4.0 International License. To view a copy of this license, visit http://creativecommons.org/licenses/by/4.0/

25 November 2022

The Vertebral Fractures Study

What is a vertebral fracture?

A vertebral fracture is a break or compression in the bones of a person's spine. Vertebral fractures are also called 'spinal fractures', 'wedge fractures', or 'compression fractures'. Your spine starts where your head meets your neck, and runs down your back. A vertebral fracture is often caused by osteoporosis, a common disease that causes bones to become thinner and break more easily. People who have had a vertebral fracture often go on to break other bones.

Why is it important to identify vertebral fractures?

When a healthcare professional identifies vertebral fractures, it is often a sign of weaker bones. Having one fracture can mean you have further spinal fractures, and other fractures. If you have been identified as having a vertebral fracture you may be offered medicine to help strengthen your bones. This medicine can lower the chances of having another fracture. However, vertebral fractures can be difficult to identify and many people go undiagnosed.

Getting a diagnosis is also important. A diagnosis can give you an explanation for your pain and can help with the management and treatment of your condition.

Who is this leaflet for?

This guide is for people who have back pain and are not sure of the reason. It will help you identify whether you have had a vertebral fracture, and if you have a fracture it will provide information to help you reduce the risk of further fractures.

How are vertebral fractures diagnosed?

Vertebral fractures can be identified in different ways:

- Patients may visit healthcare services such as their GP practice or Accident and Emergency (A&E) with symptoms that suggest they might have had a vertebral fracture. Their GP or other healthcare professional can then refer them to have an X-ray, or another kind of scan, to see if they have had a vertebral fracture.

- Other people have medical images taken for another reason and a vertebral fracture is found unexpectedly.
- People may also find out they have a vertebral fracture when they have extra images taken as part of a bone densitometry (DXA) scan as part of osteoporosis assessment to find out how strong their bones are.

What are the symptoms of vertebral fractures?

Identifying vertebral fractures can be difficult. A vertebral fracture may not cause pain, but sometimes does. It is very easy for healthcare professionals and people who have a vertebral fracture to mistake the symptoms of vertebral fractures for other problems such as a pulled muscle. Some of the symptoms of vertebral fractures can include:

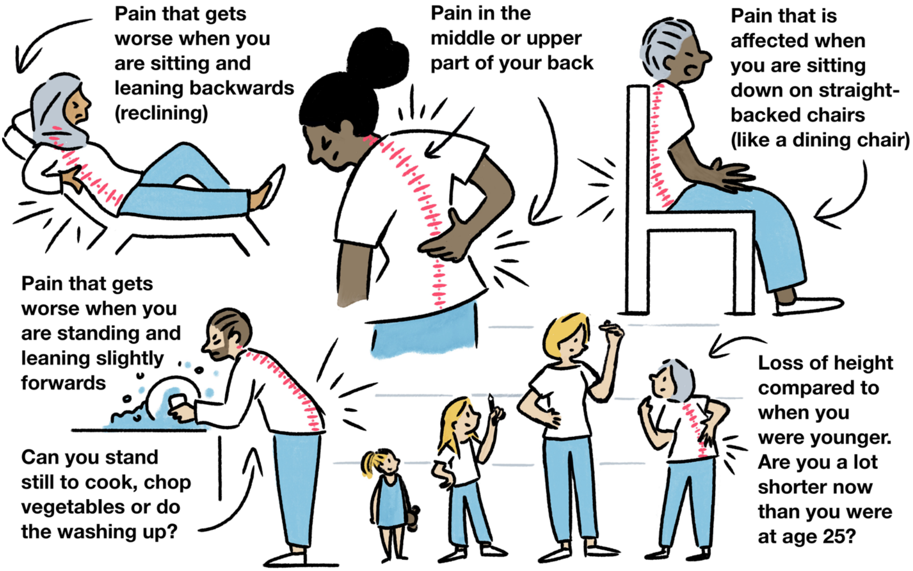

What puts people at greater chance of having vertebral fractures?

Some people are more likely to have vertebral fractures than others. It is important to note that some people who have vertebral fractures do not have any obvious risk factors, including men and younger people.

Common risk factors include:

- Aged 50 years or over
- Being a woman
- Having a parent who has had a hip fracture
- A history of breaking bones
- A diagnosis of osteoporosis
- Taking certain medications that can weaken bones. These include steroids, some anti-epileptic medications and some breast or prostate cancer treatments.
- Medical conditions including untreated premature menopause, rheumatoid arthritis, anorexia or bulimia and coeliac disease.
- Having a low body weight, or Body Mass Index (BMI) of 19 or less
- Drinking 3 or more units of alcohol per day.
- Smoking
- Having an inactive lifestyle

For more information about what makes people more likely to have osteoporosis and vertebral fractures, please see the following information: https://theros.org.uk/information-and-support/osteoporosis/causes/

What should I do if I am concerned I have had a vertebral fracture?

If you are concerned you may have had a vertebral fracture, contact a healthcare professional such as your GP. They may refer you to a hospital for imaging to confirm if you have had a vertebral fracture. A healthcare professional should then contact you to let you know the result. If you don't hear anything after several weeks, do not be afraid to contact the person who referred you for imaging to ask when to expect your results.

It is very important that you go and see a healthcare professional if you think you have the symptoms of a vertebral fracture. This is so you can take steps to reduce your chance of having further fractures. If you have more than one or two of the symptoms of vertebral fracture for a few weeks as described in this leaflet, you should contact a healthcare professional, even if your back pain is not severe.

I have been told I have a 'compression fracture'. Is this the same as a vertebral fracture?

Healthcare professionals often use different words to describe vertebral fractures. These words can include 'compression fracture' and 'wedge deformity'. If you are unsure if you have had a vertebral fracture, then please ask,

What impact will vertebral fractures have on my life?

- Having vertebral fractures affects different people in different ways. How it affects you is likely to be dependent on a number of factors including where the vertebral fracture is, and how many you have had.
- For some people, vertebral fractures will have very little impact on their lives and they will be able to carry on with their daily activities.

- For other people, vertebral fractures will cause pain and limit what they are able to do.
- Sometimes, having a vertebral fracture can affect how you feel about yourself. Talking to other people, or accessing resources provided by the Royal Osteoporosis Society might help.
- It is very important to be aware that vertebral fractures due to osteoporosis are not the same as the sort of spinal injuries that can happen after serious injuries such as a road accident, and will not cause paralysis.
- Although your vertebral fracture will heal, it won't go back to its previous shape. It will stay compressed. This can lead to changes in the shape of your spine, particularly if you have had more than one fracture.

What treatment is available for vertebral fractures?

A healthcare professional such as a GP, physiotherapist, consultant or a nurse will discuss with you how to treat your vertebral fracture. Most of the time, vertebral fractures will heal on their own. Healing will often take 3-4 months but most of the pain usually goes away after 6-8 weeks. You may find that painkillers help during this time. After 3-4 months, exercises can be very helpful to improve muscle strength and balance, posture, reduce worry about falling, and generally improve quality of life.

For more information about the types of treatment available for vertebral fractures and how to manage the pain they may cause, please see the following information: https://theros.org.uk/information-and-support/osteoporosis/living-with-osteoporosis/recovering-from-a-broken-bone/spinal-fracture

How likely is it that I will have another fracture?

People who have had a vertebral fracture are around five times more likely to have a second vertebral fracture. There is also a high chance of having another fracture such as a hip fracture. However, there are several things you can do to reduce your likelihood of having further fractures.

Can I take any medications to reduce the likelihood of having further fractures?

If you are diagnosed with a vertebral fracture, a healthcare professional can discuss if you need to take bone protection therapies to prevent future breakages. These therapies work by slowing down or stopping your bones from thinning.

If you are diagnosed with a vertebral fracture, a healthcare professional can discuss if you need to take bone protection therapies to lower the chance of future breakages.

Medications may include:

- **Bisphosphonates** help strengthen your bones, slowing down how quickly your bones break down and reduce your chance of breaking bones in the future. Examples include *alendronate, ibadronate, risedronate,* or *zoledronate*.
- **Drugs to increase bone formation and stop bone resorption** also help to strengthen your bones by slowing down how quickly your bones break down, and increasing how quickly your bones are repaired. Examples: *Romosozumab* and *denosumab*.
- **Selective oestrogen receptor modulators (SERMs)** also help to maintain bone density and reduce the chance of further fractures. For example, *raloxifene*.
- **Parathyroid hormone** is produced in your body by the thyroid gland. Parathyroid hormone can increase bone density. For example, *teriparatide*.
- **Hormone Replacement Therapy (HRT)** are medicines that are similar to the hormones oestrogen and progesterone. HRT can be used to manage symptoms of the menopause and also strengthen bones.
- **Testosterone treatment** can be useful for men with osteoporosis, where male sex hormone levels are low. Testosterone treatment can help to slow bone loss.

Ask your GP or other healthcare professional about which medications may be right for you.

In addition to medicines to reduce the chance of having further fractures, calcium and vitamin D supplements may be recommended.

Some people may also have a vertebral fracture when they are already taking these medicines. If this happens, a healthcare professional may need to review your medicine to check that you are on the right one for you.

Your healthcare professional may also want to refer you for further tests to find out if you have osteoporosis.

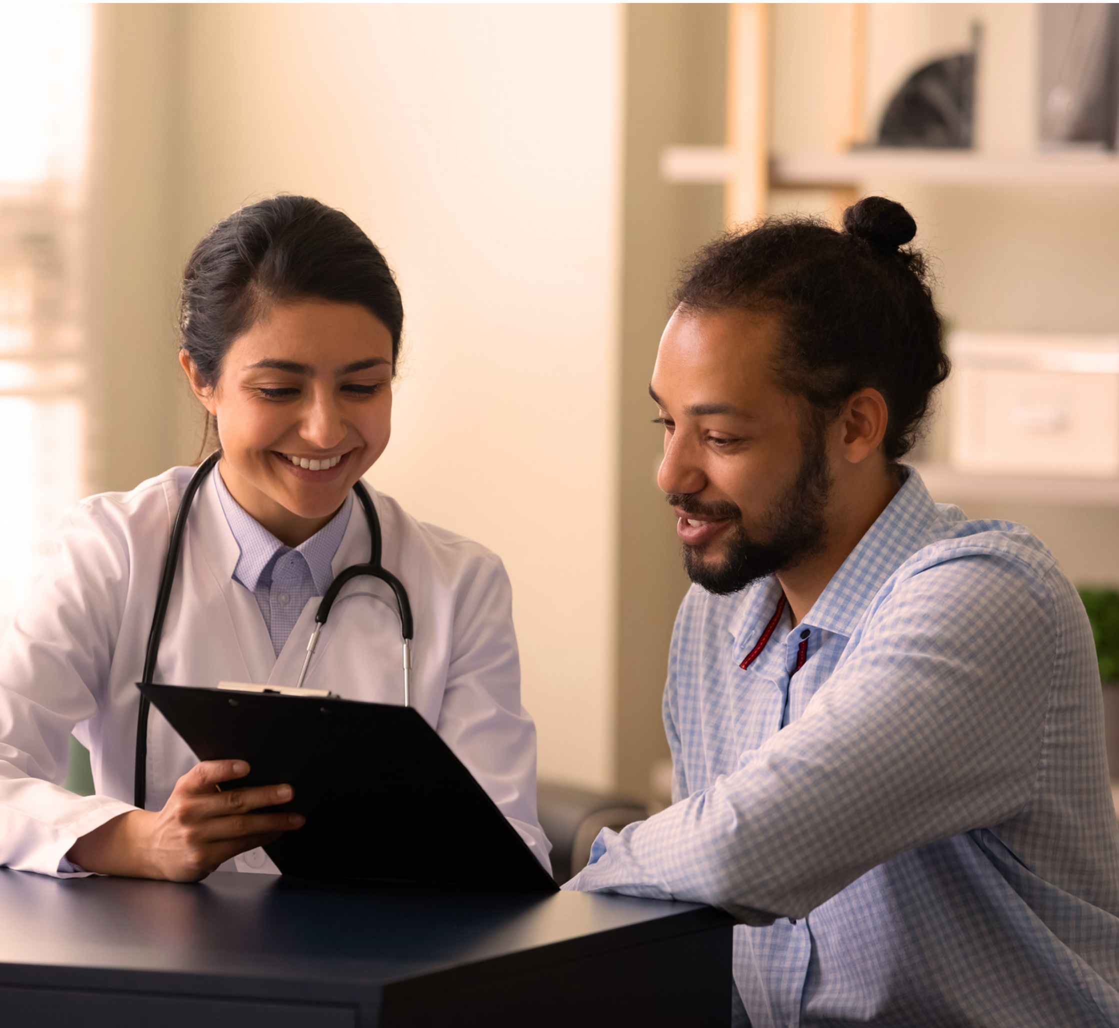

Is there anything else I can do to reduce the chance of having further fractures? Yes! You can strengthen your bones by:

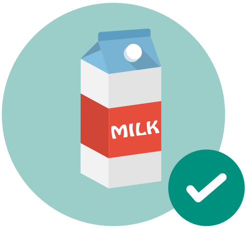

Eating foods rich in calcium and vitamin D: Calcium can be found in dairy foods like milk and cheese, and in leafy green vegetables, nuts and seeds. Vitamin D is needed for the body to use calcium, and can be found in fish like salmon, tuna and sardines. Your doctor may recommend you take Calcium and Vitamin D supplements.

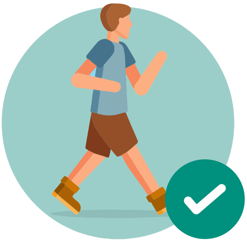

Exercising regularly: Regular weight-bearing exercise is important to keep your bones healthy. More information about exercises is provided overleaf.

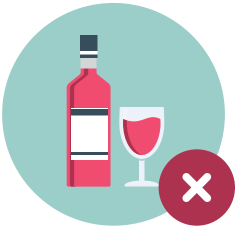

Avoid drinking too much alcohol: Drinking a lot of alcohol can decrease the density of your bones, making them more prone to breaking. The NHS recommends not drinking more than 14 units of alcohol in a week.

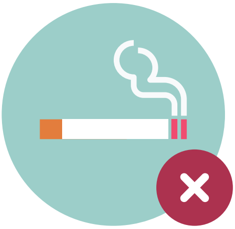

Stopping smoking: Smoking can increase the likelihood of osteoporosis.

Should I avoid exercises to avoid having another fracture?

The thought of getting another fracture might be worrying, but exercise and keeping moving are some of the best things you can do to reduce the chance of more broken bones. Activity is unlikely to cause any further damage to your spine. Activity is great for bone health and osteoporosis - whatever your age or wellness.

Begin with activities you know you can do comfortably. Gradually, over time, increase the amount and intensity to reach your target. You'll find that 'little and often' is best to begin with.

Warming up is important. This could be walking on the spot. If you are used to exercises already then you may already have ways to warm up, such as marching. A factsheet is available from the Royal Osteoporosis Society called *'Before and After Exercise'*: https://tinyurl.com/BeforeAfterExercise.

Exercises that are good for bone health and osteoporosis include those that help with pain, posture, bone and muscle strength, and exercises that improve balance and reduce falls. Everyone who has had an osteoporotic vertebral fracture is encouraged to gradually increase the amount of activity and exercise they do. Factsheets on all these types of exercises are available from the Royal Osteoporosis Society.

We know that finding out you have had a vertebral fracture can be difficult. Your healthcare professionals are there to support you and there are some things you can do to help yourself. We hope that this booklet will answer some of your questions and provide you with information to help prevent you from having more fractures in the future.

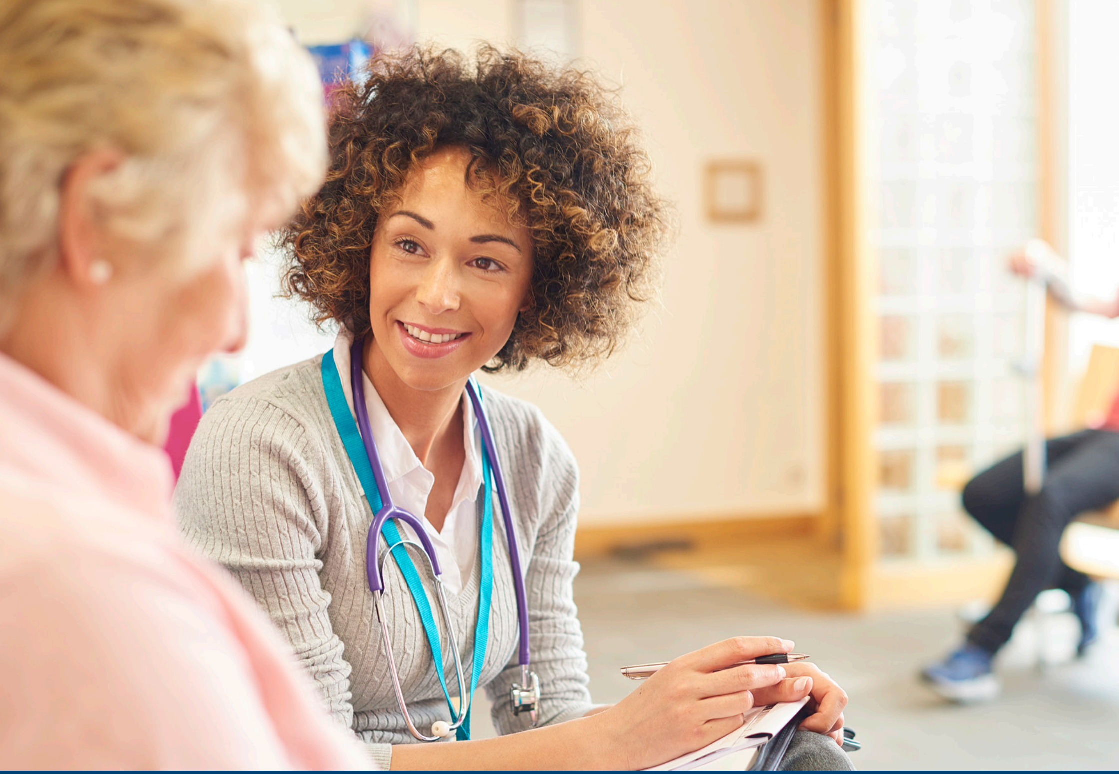

Where else can I find reliable information about osteoporosis and vertebral fractures?

The Royal Osteoporosis Society provides information and advice about osteoporosis and bone health:

www.theros.org.uk

You can also get help and support from a Specialist Nurse by calling their free helpline:

0808 800 0035

University of BRISTOL

FUNDED BY NIHR | National Institute for Health Research

The Vertebral Fractures Study
