# Supplementary material for: Improving patients’ experiences of diagnosis and treatment of vertebral fracture: co-production of knowledge sharing resources
Source: BMC Musculoskelet Disord. 2024 Feb 21;25:165. doi: 10.1186/s12891-024-07281-9 (PMC10880218; doi:10.1186/s12891-024-07281-9)
Supplement: Supplementary file 6 — Additional file 6 [file 12891_2024_7281_MOESM6_ESM.pdf]

Spinal fracture:
Breaks in the bones of your spine

Neck or back pain?
Could you have a vertebral spinal fracture?

What is a vertebral fracture and why are they important?

A vertebral fracture is a break or compression in the bones of a person's spine. Vertebral fractures are also called 'spinal fractures', 'wedge fractures', or 'compression fractures'.

Vertebral fractures are often caused by osteoporosis, a disease that causes bones to become thinner and break more easily.

If you have a vertebral fracture you may be offered medicine to help strengthen your bones. This medicine can lower the chances of having another fracture.

What are the symptoms of vertebral fractures?

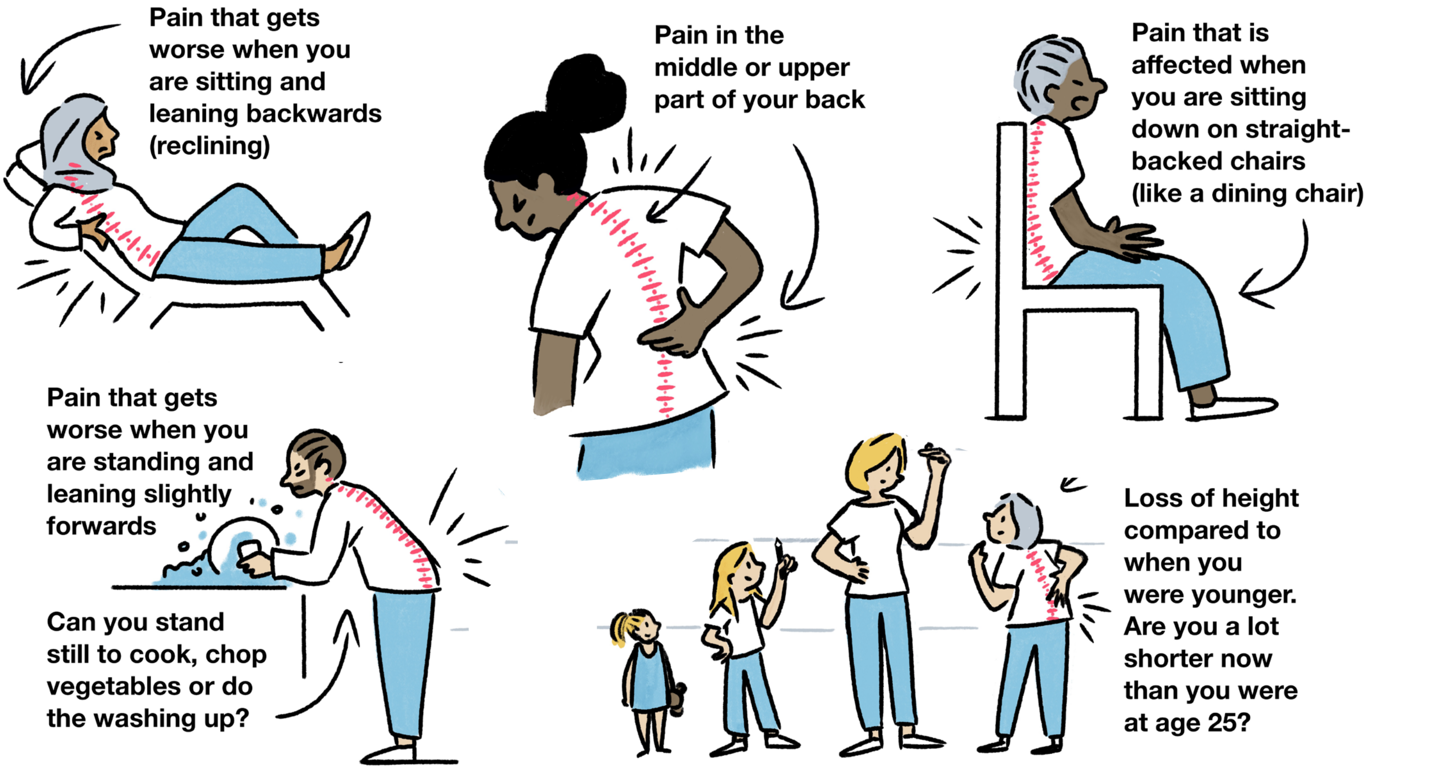

If you have more than one or two of the symptoms of vertebral fracture for a few weeks contact a healthcare professional, even if your back pain is not severe.

For more information on the diagnosis of vertebral fractures, please see our extended guide https://tinyurl.com/Diagnosis-for-patients

Funding: This project is funded by the NIHR Research for Patient Benefit programme, NIHR201523. The views expressed are those of the authors and not necessarily those of the NIHR or the Department of Health and Social Care.

University of BRISTOL

FUNDED BY NIHR | National Institute for Health Research

The Vertebral Fractures Study
